# A comparative study of simulation-based inference methods for epidemic models with identifiability considerations

**DOI:** 10.1371/journal.pcbi.1014364

**Published:** 2026-06-02

**Authors:** Geunsoo Jang, K. Selçuk Candan, Gerardo Chowell

**Affiliations:** 1 School of Computing and Augmented Intelligence, Arizona State University, Tempe, Arizona, United States of America; 2 Department of Population Health Sciences, School of Public Health, Georgia State University, Atlanta, Georgia, United States of America; 3 Department of Applied Mathematics, Kyung Hee University, Yongin, Korea; University of Utah, UNITED STATES OF AMERICA

## Abstract

Epidemic models play a critical role in understanding transmission dynamics, generating forecasts, and informing public health interventions when they are properly calibrated to epidemiological data. Traditional Bayesian inference methods rely on the likelihood function to update prior knowledge using observed data. However, for realistic epidemic models, likelihood functions are often analytically intractable or computationally prohibitive, which can limit the applicability of these methods. Simulation-based inference provides a promising alternative by approximating posterior distributions through forward simulations rather than an explicit likelihood evaluation. In this study, we present a systematic comparison of four approaches: Approximate Bayesian Computation (ABC), Neural Posterior Estimation (NPE), a neural method with temporal embedding, and Preconditioned Neural Posterior Estimation (PNPE), which integrates elements of both classical and neural techniques. These methods are evaluated across epidemic models of increasing complexity under fixed simulation budgets and varying levels of observational noise, with explicit attention to both structural and practical identifiability. Our results show that neural methods generally improve posterior fidelity and predictive accuracy compared with ABC under constrained simulation budgets. PNPE achieved strong performance in several simulation settings, whereas temporal embeddings improved inference in models with complex epidemic dynamics by capturing sequential dependencies. These gains come with important trade-offs: PNPE required substantially greater computational resources and, unlike fully amortized NPE-based methods, may require reconditioning for each new observation. In contrast, ABC remained computationally efficient and provided reasonable, though often more conservative, posterior estimates. Overall, our findings highlight trade-offs among computational efficiency, posterior accuracy, uncertainty calibration, and inference reusability, suggesting that method selection should depend on model complexity, data quality, identifiability, and available computational resources.

## Author summary

We developed a systematic framework to compare methods for estimating parameters in epidemic models. These models help researchers understand how diseases spread and how interventions may change the course of an outbreak. However, estimating model parameters can be difficult because traditional likelihood-based methods are often impractical for realistic epidemic models. Simulation-based inference offers an alternative by generating synthetic epidemic trajectories and comparing them with observed data. In this study, we compared four inference methods, including both classical and neural network-based approaches. We tested these methods across epidemic models with different levels of complexity, observational noise, and identifiability. Identifiability is important because it determines whether the available data contain enough information to estimate model parameters reliably. Our results highlight the strengths and limitations of each method in terms of accuracy, uncertainty quantification, and computational cost. These findings provide practical guidance for selecting inference methods and for using epidemic models in forecasting and decision-making during future outbreaks.

## 1. Introduction

Understanding the dynamics of infectious diseases is essential for public health decision-making, especially during emerging epidemics. Mechanistic epidemic models, such as compartmental models [1–3], provide a principled framework for capturing transmission dynamics and evaluating the impact of interventions. Accurate and timely parameter inference for these models not only supports short-term forecasting but also guides decisions about resource allocation, intervention planning, and risk communication. The COVID-19 pandemic made this need especially clear, highlighting the importance of flexible and computationally efficient inference methods capable of handling data scarcity, uncertainty, and rapidly evolving epidemic conditions. However, drawing reliable inferences remains challenging due to statistical complexities and heavy computational demands.

Bayesian inference offers a rigorous framework for quantifying uncertainty in model parameters given the observed data. At its core, Bayesian inference relies on the likelihood function to update prior beliefs into a posterior distribution using Bayes’ theorem [4]. However, many realistic epidemic models lead to intractable or computationally expensive likelihood functions due to latent variables, complex observation processes, or stochastic dynamics [5–7]. Such features make it difficult to specify an analytical likelihood or evaluate it efficiently for each parameter draw. Consequently, traditional likelihood-based methods such as Markov chain Monte Carlo (MCMC [8]) may become computationally prohibitive in practice. To address these challenges, various likelihood-based approaches have been developed, including data-augmented MCMC, partially observed Markov process (POMP) formulations, iterated or particle filtering approaches, and particle MCMC methods [7,9–11]. Although these methods provide principled statistical inference, they typically involve substantial computational costs and may not scale well to high-dimensional or complex models. These limitations have motivated the use of simulation-based approaches that can leverage forward simulations without requiring an explicit likelihood.

### 1.1. Simulation-based inference

Simulation-based inference (SBI), also known as likelihood-free inference, provides a powerful alternative by leveraging the forward simulations of a model without requiring an explicit likelihood function [12]. The posterior approximation is achieved by comparing simulated data with observed data, either through summary statistics or with the aid of neural density estimators. SBI methods have been widely applied across diverse scientific domains and represent an active area of methodological development. Applications span a broad range of fields, including particle physics, cosmology, astroparticle physics, and computational biology, where likelihood-free approaches are essential for modeling complex and high-dimensional systems [13–16].

SBI methods have been increasingly applied in the context of epidemic models [17–23]. Approximate Bayesian Computation (ABC [24]) has been used to fit complex models where traditional likelihood-based inference is infeasible. McKinley et al. (2018) have provided a comprehensive review of some of the more popular variants of ABC for complex epidemic systems, illustrating their application to high-dimensional, computationally intensive transmission models [17]. In a complementary tutorial, Minter and Retkute (2019) introduced ABC for infectious disease modeling, discussed user-defined choices, such as summary statistics, tolerance schedules, and perturbation kernels, and demonstrated rejection-ABC and sequential Monte Carlo ABC (SMC-ABC) implementations in R across three case studies—including deterministic SIR, age-structured stochastic measles, and spatial individual-based models—highlighting both the flexibility and computational trade-offs of ABC approaches [18].

More recently, neural network–based SBI methods have been proposed to overcome the limitations of ABC in high-dimensional settings. These approaches, which are often referred to as neural density estimators or amortized inference methods, learn flexible mappings from simulated data to posterior distributions. Radev et al. (2021) introduced OutbreakFlow, which leverages neural posterior estimation to infer the parameters of compartmental models in real time during outbreaks [19]. Wood et al. (2022) demonstrated how probabilistic programming can automate inference tasks in existing stochastic epidemiological simulators, effectively decoupling model specifications from inference [20]. Arnst et al. (2022) applied a machine-learning simulation-based inference approach to identify the parameters of a hybrid stochastic COVID-19 model in a university campus setting [23]. These neural approaches promise improved scalability, efficiency, and flexibility compared to traditional ABC, especially for complex or partially observed epidemic models. Recent innovations in this field have expanded the utility of amortized SBI to handle more realistic challenges, such as missing or incomplete observational data [25,26]. Furthermore, while much of the literature focuses on deterministic models, recent work has demonstrated the efficacy of NPE for stochastic epidemic modeling, providing robust inference even under inherent system noise [27].

Taken together, these studies demonstrate the methodological maturity and practical relevance of ABC and SBI in epidemic modeling. However, most applications focus on a single model structure or a single inference algorithm and rarely evaluate multiple SBI methods simultaneously under varying conditions of identifiability and noise. Moreover, as these methods move toward real-world deployment, identifying model misspecification—where the simulator fails to capture the true data-generating process—has become a critical diagnostic requirement [28]. Other emerging research explores hybrid strategies that bridge classical ABC with neural density estimation to balance computational efficiency with approximation accuracy [29].

Despite these advancements, identifiability issues are often neglected in the SBI literature, despite their strong influence on posterior behavior and predictive accuracy. We distinguish between structural identifiability, which concerns the uniqueness of parameters based on the model’s mathematical formulation, and practical identifiability, which assesses estimation precision given the limitations of noisy, real-world data. Both forms of identifiability present persistent challenges for epidemiological estimation [30,31]. This motivates our study’s systematic comparison of several SBI approaches under a unified experimental framework that explicitly incorporates identifiability considerations and robustness analyses.

### 1.2. Our contributions

In this paper, we make two contributions. First, we conduct what is, to our knowledge, the first comparative evaluation of four SBI methods—ABC [24], neural posterior estimation (NPE) [32,33], NPE using a Long Short-Term memory embedding network (NPE-LSTM), and the recently proposed preconditioned NPE (PNPE) [34]—in the context of epidemic modeling. We apply these methods to a suite of models ranging from the classical Susceptible-Exposed-Infected-Recovered (SEIR) framework to more complex disease-specific structures such as the Ebola model [1–3,35].

Second, we establish a benchmarking framework that explicitly integrates identifiability analysis to assess SBI robustness under realistic epidemiological conditions. Specifically, we consider both structural and practical identifiability and examine how these properties interact with posterior approximation, predictive calibration, and simulation budget constraints. By embedding identifiability into the evaluation pipeline, our study provides a systematic analysis of how SBI methods behave when parameter recoverability is fundamentally limited by the model structure or degraded by observational noise.

Unlike prior general-purpose SBI benchmarks [16,36] that emphasize performance across heterogeneous simulators, our objective is domain-specific and theoretically grounded. Mechanistic epidemiological ODE models exhibit intrinsic nonlinear parameter couplings, strong temporal dependencies, and potential structural non-identifiability that fundamentally alter posterior geometry and predictive behavior. These characteristics are rarely examined explicitly in existing SBI benchmarking studies. By focusing on identifiability-aware validation in dynamical epidemic systems, our framework complements prior benchmarking efforts and addresses a practically critical yet underexplored dimension of SBI evaluation.

Overall, these contributions establish one of the first comprehensive, identifiability-aware benchmarks of SBI methods for epidemic modeling. By clarifying their relative strengths and limitations under realistic epidemiological constraints and highlighting the central role of identifiability, our study provides empirically grounded guidance for the reliable use of SBI in future epidemic response and preparedness efforts.

## 2. Results

In this section, we present results from three epidemic models of increasing complexity: the classical SEIR model [3], the Ebola model [2], and spatially informed rapid testing for epidemic modeling (SIRTEM) [35]. We begin with the SEIR and Ebola models, and finally the SIRTEM framework. For each model, we compare the performance of ABC [24], NPE [32,33], NPE-LSTM [19,37], and PNPE [34], examining posterior distributions, predictive accuracy, and robustness under different identifiability regimes. The prior distributions and ground-truth parameter set used for all three models are summarized in Tables A-C in S1 Text.

For the SEIR and Ebola models, we additionally obtain reference posteriors using MCMC, which serve as a baseline for assessing posterior accuracy and calibration. In these experiments, we run 4 independent MCMC chains with 5,000 iterations each, discard the initial warm-up samples, and retain 10,000 post-warm-up posterior samples per dataset. Convergence is assessed using standard diagnostics, including trace plots, effective sample size, potential scale reduction factors, and divergent-transition checks. Due to the high computational cost of MCMC sampling, particularly for models with increasing dimensionality and complexity, it is not applied to the SIRTEM model.

### 2.1. Posterior estimation

**Fig 1:**
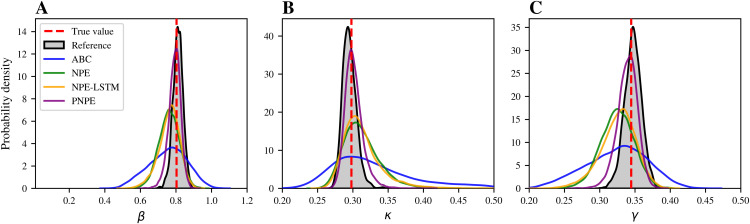
Posterior distributions of the SEIR model parameters. **A** β, **B** κ, and **C** γ, estimated by ABC (blue), NPE (green), NPE-LSTM (orange), and PNPE (purple). The red dashed line indicates the true parameter value. The gray shaded distribution with a black outline indicates the reference MCMC posterior.

In Fig 1, we compare the posterior distributions of the SEIR model parameters estimated by ABC, NPE, NPE-LSTM, and PNPE. The estimated parameters include the transmission rate (β), the progression rate from exposed to infectious individuals (κ), and the recovery rate (γ). For all three parameters, PNPE consistently yielded posterior estimates that were more concentrated around the ground truth. In contrast, the ABC posteriors were markedly wider, particularly for κ and γ.

To comprehensively evaluate the performance of each inference method, we conducted posterior predictive checks (PPC) and reported quantitative metrics, including mean squared error (MSE), mean absolute error (MAE), weighted interval score (WIS), 95% predictive interval coverage (PI), maximum mean discrepancy (MMD), classifier 2-sample test (C2ST), and runtime, as summarized in Table D in S1 Text. PNPE showed the closest agreement with the reference posterior based on both the MMD (0.01 ±0.02) and C2ST (0.55 ±0.03). In addition, PNPE achieved the lowest MSE (1011.35 ±206.34) and MAE (18.30 ±2.06) values, indicating that its predictive trajectories remained closest to the observed data among the evaluated methods. PNPE also achieved the lowest WIS (42.08 ±4.44) while maintaining a 95% PI (96.90 ±1.30) close to the nominal 95% level, suggesting a favorable balance between predictive concentration and uncertainty calibration. In contrast, ABC produced higher 95% PI (98.40 ±1.20) together with substantially larger WIS values (49.41 ±12.11), indicating broader and more conservative predictive intervals. Nevertheless, ABC remained the most computationally efficient method, with the lowest runtime (31.08 ± 7.19 seconds), while still providing competitive predictive performance. Although NPE and NPE-LSTM enable near-instantaneous inference after training, their total wall-clock runtimes were longer than that of the reference posterior in the SEIR experiment due to substantial upfront training costs, highlighting the practical trade-off between training cost and inference speed.

**Fig 2:**
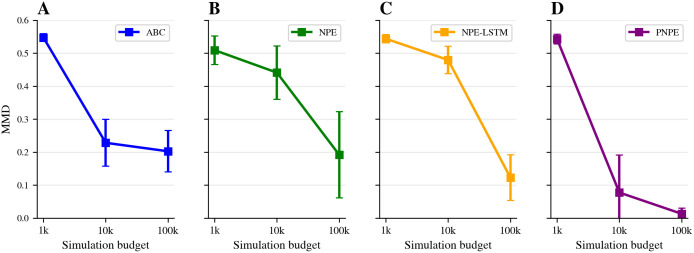
Maximum mean discrepancy (MMD) between each method’s posterior and the MCMC reference posterior under the SEIR model for different simulation budgets (1k, 10k, and 100k). **A** ABC, **B** NPE, **C** NPE-LSTM, and **D** PNPE. Each point represents the mean MMD computed over 10 independent observed datasets, and error bars indicate the standard deviation. Lower MMD values indicate closer agreement between the inferred posterior and the reference posterior.

Fig 2 illustrates the MMD between the inferred posteriors and the reference as the simulation budget increases (1k, 10k, and 100k). All methods show improved agreement with the reference posterior as the simulation budget increases. Among the evaluated methods, PNPE consistently achieves the lowest MMD values across all budgets, indicating closer agreement with the reference posterior distribution. At 100k simulations, PNPE nearly converges to the reference posterior, whereas ABC remains substantially distant even with increased data. Although sharper posterior concentration may reflect overconfidence, the combined evaluation using MMD, C2ST, WIS, and predictive interval coverage indicates that PNPE achieves the strongest overall performance among the evaluated SBI methods, at the expense of substantially higher computational cost. NPE-LSTM also performs well, striking a balance between accuracy and efficiency.

Tables E and F in S1 Text show the quantitative predictive performance and runtime results for the Ebola and SIRTEM models, and the corresponding posterior distributions are presented in Figs 1 and 2 in S1 Text. As these models generate multiple outputs, performance was evaluated by jointly considering posterior predictive error across all outputs. In the SIRTEM model, ABC and PNPE showed competitive point-prediction performance. However, their higher WIS values suggest less favorable uncertainty quantification, indicating that the predictive intervals may not adequately capture uncertainty in this setting. In contrast, NPE and NPE-LSTM often achieved lower WIS values, reflecting better uncertainty calibration in this setting. Notably, unlike the simpler SEIR model, NPE-LSTM showed comparable or better performance relative to NPE in both the Ebola and SIRTEM models, suggesting that sequence-based architectures offer additional advantages in more complex settings. Table G in S1 Text presents the computational time required to generate the simulation data across different models (SEIR, Ebola, and SIRTEM) and simulation budgets (1k, 10k, and 100k). The computational cost increases approximately linearly with the number of simulations across all models. Among the evaluated models, the SIRTEM model requires substantially greater computational resources due to its increased structural and dimensional complexity.

### 2.2. Impact of identifiability

Identifiability refers to whether model parameters can be uniquely inferred from the observed data. Structural identifiability concerns the theoretical uniqueness of parameter recovery under ideal conditions. A model is structurally non-identifiable when multiple parameter combinations produce indistinguishable epidemic trajectories. Meanwhile, practical identifiability reflects whether parameters can be reliably estimated in the presence of observational noise and limited data.

We characterized structurally identifiable and non-identifiable regimes for the SEIR and Ebola models using the DAISY software [38], as summarized in Table H in S1 Text. For the SEIR model, all parameters are structurally identifiable when the initial conditions are known. In contrast, when the initial conditions are unknown, the transmission rate (β) and total population size (*N*) become structurally non-identifiable. This occurs because the infection dynamics depend on the combined term βSI/N, such that multiple combinations of β and *N* can produce indistinguishable epidemic trajectories.

For the Ebola model, the structural identifiability properties depend strongly on the available observation data. When newly infected individuals (κE), hospitalized individuals (αI), and deaths ($\delta_{I}I+\delta_{H}H$) are jointly observed, all model parameters are structurally identifiable. In contrast, when death observations are excluded, several hospitalization and transmission-related parameters, including $\beta_{H}$, $\delta_{H}$, $\gamma_{H}$, $\beta_{I}$, $\delta_{I}$, and $\gamma_{I}$, become structurally non-identifiable. This non-identifiability arises because, without death observations, the downstream dynamics of hospitalized individuals cannot be uniquely disentangled. As a result, multiple combinations of transmission, recovery, and mortality parameters can produce indistinguishable epidemic trajectories.

**Fig 3:**
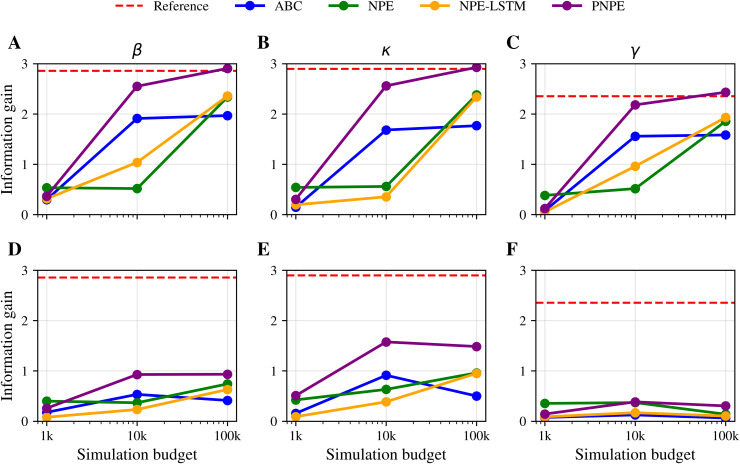
Information gains across simulation budgets and identifiability conditions under the SEIR model. **A** Structurally identifiable and β, **B** Structurally identifiable scenario and κ, **C** Structurally identifiable scenario and γ, **D** Structurally non-identifiable scenario and β, **E** Structurally non-identifiable scenario and κ, and **F** Structurally non-identifiable scenario and γ. Information gain is reported for ABC (blue), NPE (green), NPE-LSTM (orange), and PNPE (purple) as a function of the simulation budgets (1k, 10k, and 100k). The dashed red line indicates the information gain between the reference posterior and the prior.

To quantitatively assess how the simulation budget and model identifiability influence the precision of posterior estimates, we analyzed the information gain across different regimes, as shown in Fig 3. Information gain was computed as the marginal Kullback–Leibler (KL) divergence between the inferred posterior and the prior for each parameter. Thus, the metric measures how much the marginal posterior distribution differs from the prior distribution after inference. An increase in this metric indicates that additional simulations contribute to a larger difference between the prior and the posterior. However, a higher information gain does not necessarily indicate that the parameters are accurately estimated, as it may reflect posterior over-concentration rather than genuine parameter recovery.

In the structurally identifiable scenario (Figs 3A-C), all inference methods demonstrated increasing posterior updating with the simulation budget. This trend suggests that additional simulations allow the methods to extract more information from the simulated data and move the posterior away from the prior when the parameters are structurally identifiable. Among the compared methods, PNPE achieved the highest information gain across most parameters and budgets, indicating that PNPE produced the strongest posterior update relative to the prior in these identifiable settings. At a simulation budget of 100k, PNPE achieved information gain values of 2.91, 2.93, and 2.43 for β, κ, and γ, respectively, closely approaching the reference posterior. These results are consistent with the posterior and predictive performance reported in Fig 1 and Fig 2. However, because higher information gain can also reflect posterior over-concentration, we interpret the information gain only in combination with the reference comparison, predictive accuracy, WIS, and coverage. In the structurally identifiable SEIR setting, these complementary metrics suggest that PNPE closely approximates the reference posterior at larger simulation budgets.

In contrast, under structurally non-identifiable conditions (Figs 3D-F), the information gain is substantially lower and less consistent than in the structurally identifiable scenarios. Across all methods, the information gain remains well below the reference level, indicating that the posterior distributions do not move far from the prior even as the simulation budget increases. This behavior is consistent with the theoretical limitation imposed by structural non-identifiability, where multiple parameter combinations can generate indistinguishable epidemic trajectories.

The effect of increasing the simulation budget is not uniform across parameters or methods. For β and γ (Figs 3D and F), most methods show only modest changes in information gain, and the values remain low across all budgets. For κ (Fig 3E), some methods, particularly PNPE and NPE-LSTM, show relatively larger information gain at higher simulation budgets compared with β and γ. This is likely because κ directly enters the observation process through the incidence (κE), so the observed data contain more direct information about κ than about the other parameters.

Tables I and J in S1 Text summarize the information gain for the SEIR and Ebola models, respectively. Consistent with the findings in the SEIR model, the Ebola model also shows that, under structurally non-identifiable conditions, the information gain remains limited and does not consistently increase as the simulation budget increases. Although some parameters may show partial posterior updating when they are more directly constrained by the observation process, the overall information gain remains substantially lower than in structurally identifiable settings. This pattern further supports the interpretation that, in the absence of structural identifiability, additional simulations alone are not sufficient to ensure reliable or unique parameter recovery.

To evaluate practical identifiability under realistic observational uncertainty, we performed a noise-sensitivity experiment. We considered Poisson noise as a baseline observation model and negative binomial noise to represent overdispersed epidemic data. For the negative binomial model, we varied the dispersion parameter *r*, with smaller values corresponding to stronger observational noise. We treated *r* = 50 as a moderate noise condition and *r* = 10 as a high noise condition. We compared the posterior predictive intervals and quantitative performance metrics across inference methods to examine how increasing observational noise affects practical identifiability.

**Fig 4:**
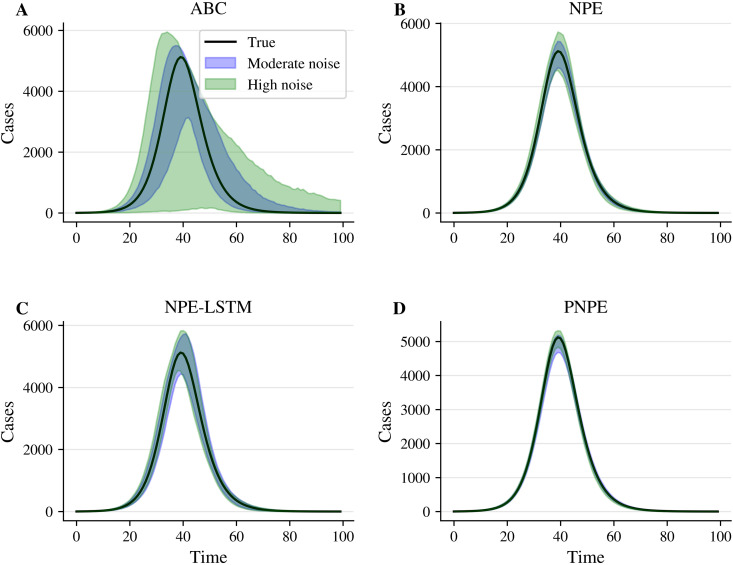
Posterior predictive simulation results for the SEIR model under different observational noise conditions. **A** ABC, **B** NPE, **C** NPE-LSTM, and **D** PNPE. The black curve represents the true epidemic trajectory. Shaded regions indicate the 95% predictive intervals obtained from posterior predictive simulations using samples drawn from the inferred posterior distributions. The blue and green shaded regions denote 95% posterior predictive intervals under negative binomial observation noise with dispersion parameters *r* = 50, corresponding to moderate noise, and *r* = 10, corresponding to high noise, respectively.

Fig 4 presents posterior predictive intervals of the SEIR model under different observational noise levels. The black curve indicates the true epidemic trajectory, and the shaded regions represent the 95% PI under moderate and high noise conditions. Overall, predictive uncertainty increases as observational noise increases, indicating that practical identifiability deteriorates as data become noisier. For ABC (Fig 4A), the 95% PI is noticeably wider than those of the neural inference methods, particularly under the high noise condition. The high noise interval also exhibits a broader right tail, indicating greater uncertainty in the timing and magnitude of the epidemic peak. The corresponding quantitative evaluation, including MSE, MAE, and WIS, is summarized in Table K in S1 Text. Under the moderate noise condition (negative binomial with *r* = 50), ABC yielded substantially higher MSE, MAE, and WIS values than the neural inference methods. For example, the WIS was 293.94 ±134.53 for ABC, compared with 75.01 ±11.26, 79.87 ±10.90, and 63.83 ±10.55 for NPE, NPE-LSTM, and PNPE, respectively. Under the higher noise condition (*r* = 10), ABC again showed the largest WIS value of 368.35 ±124.34.

In contrast, NPE, NPE-LSTM, and PNPE (Figs 4B-D) produce substantially narrower predictive intervals that closely follow the true epidemic trajectory. These methods maintain relatively well-centered predictions even when the noise level increases, as summarized in Table K in S1 Text. In the SEIR model, PNPE achieved the lowest WIS under Poisson noise and moderate negative binomial noise, while NPE and NPE-LSTM showed competitive performance under high noise.

The quantitative results for the Ebola model, summarized in Table L in S1 Text, show a similar trend. Across most cases and noise settings, neural SBI methods achieved lower MSE, MAE, and WIS values than ABC. For example, in Case 2 under moderate noise (*r* = 50), ABC had a WIS of 74.35, whereas NPE, NPE-LSTM, and PNPE achieved lower WIS values of 37.54, 38.15, and 35.42, respectively. These results suggest that the improved predictive performance of neural SBI methods extends beyond the SEIR model to the more complex Ebola model.

### 2.3. Real-data application

**Fig 5:**
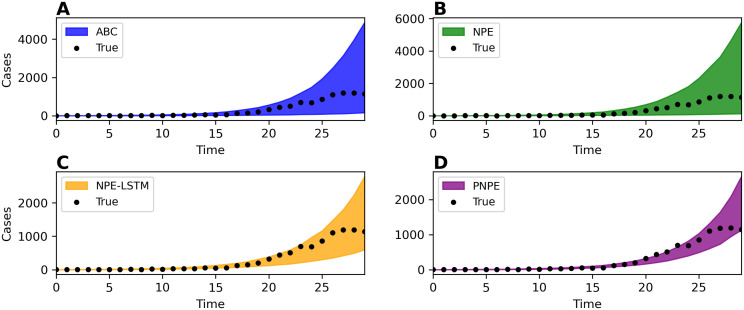
Posterior predictive simulation for the 1918 San Francisco influenza data using different inference methods. **A** ABC, **B** NPE, **C** NPE-LSTM, and **D** PNPE. Black dots indicate the observed incidence data. Shaded regions indicate the 95% posterior predictive intervals obtained by simulating trajectories from posterior samples inferred by each method.

We further evaluate the posterior predictive performance using real epidemic data with a simulation budget of 10k, as shown in Fig 5. We use the 1918 San Francisco influenza dataset analyzed in [39] and adopt the same initial conditions and prior distributions as specified in that study to ensure consistency in the experimental setup. In this real-data setting, the model is subject to practical identifiability constraints arising from observation noise and limited data, making it a more realistic and challenging inference scenario. Across all methods, the predicted trajectories generally capture the overall increasing trend of the observed cases, indicating that each inference approach can recover the main epidemic dynamics from the data. However, quantitative differences emerge in predictive accuracy and uncertainty calibration, as summarized in Table M in S1 Text.

ABC produces substantially wider 95% PI, particularly during the later stages of the epidemic, resulting in 100% predictive interval coverage but relatively large WIS values (199.11), indicating broad and conservative uncertainty estimates. In contrast, NPE-LSTM and PNPE generate more concentrated 95% PI and achieve lower MSE and WIS values than ABC. However, both PNPE and NPE-LSTM yield predictive interval coverage below the nominal 95% level (86.67%), suggesting potential undercoverage and overconfident uncertainty estimates despite their improved predictive accuracy. NPE-LSTM offers a more favorable balance by maintaining competitive predictive accuracy while achieving the lowest WIS among the evaluated methods. These findings suggest that no single SBI method is uniformly optimal across all settings, and that the choice of inference method should depend on the desired balance between predictive precision and reliable uncertainty quantification.

## 3. Discussion

In this study, we systematically evaluated SBI methods (ABC, NPE, NPE-LSTM, and PNPE) across three epidemiological models with increasing complexity (SEIR, Ebola, and SIRTEM).

### 3.1. Predictive accuracy and uncertainty calibration

Our comparison framework combined quantitative metrics (MSE, MAE, WIS, 95% PI coverage, and runtime), MMD, and C2ST to assess both the predictive accuracy and uncertainty calibration under varying identifiability and noise conditions. In particular, these metrics provide complementary perspectives: point-wise error metrics (MSE, MAE) assess predictive accuracy; WIS and coverage evaluate uncertainty calibration; and MMD and C2ST capture discrepancies in the overall posterior distribution. The results highlight the complementary roles of the evaluation metrics reported in Tables D-F in S1 Text.

MSE and MAE primarily assess the accuracy of the predictive mean but do not capture the uncertainty. In contrast, WIS and 95% PI coverage evaluate the reliability and calibration of PI, providing insight into uncertainty quantification. Meanwhile, MMD and C2ST measure the discrepancy between the inferred and reference posterior distributions, offering a distribution-level assessment of posterior fidelity. Taken together, these metrics provide a more comprehensive evaluation of inference performance.

Across the evaluated models, neural SBI methods generally improved predictive accuracy and posterior agreement relative to ABC under fixed simulation budgets. However, these gains were accompanied by important calibration trade-offs. ABC was computationally efficient and often captured the overall epidemic trajectory, but it tended to yield broader predictive intervals and higher WIS values. Neural SBI methods produced sharper posterior distributions that more closely matched the reference posterior in the SEIR and Ebola experiments, but sharper estimates did not always imply better calibration. In particular, the real-data experiment showed reduced 95% PI coverage for PNPE and NPE-LSTM, suggesting potential undercoverage.

While LSTM-based embeddings can be beneficial, NPE-LSTM does not universally outperform standard NPE across all settings. We observed a clear performance trade-off associated with LSTM-based embeddings. In simpler models such as SEIR, NPE-LSTM was slightly less accurate than NPE, suggesting that the high representational capacity of LSTM may be unnecessary for parsimonious systems and can lead to over-parameterization and optimization challenges. Additionally, compressing relatively simple trajectories into a fixed low-dimensional embedding (e.g., 30 dimensions) may introduce an information bottleneck, potentially discarding features that standard NPE can capture through direct conditioning. In contrast, for more complex models such as Ebola, which exhibit richer latent dynamics, NPE-LSTM showed performance comparable to or better than standard NPE in both point and interval accuracy. This indicates that sequence-aware architectures are particularly beneficial when capturing complex temporal dependencies is essential for accurate inference.

### 3.2. Computational trade-offs

Comparing the computational efficiency of the SBI methods revealed a clear trade-off between training cost, inference speed, and predictive performance. ABC did not require a training stage and was the fastest method for a single observed dataset. Despite its simplicity, ABC showed reasonable predictive performance in several settings. However, because ABC is not amortized, the inference procedure must be repeated for each new observed dataset.

By contrast, NPE and NPE-LSTM required a more expensive upfront training stage, but once trained, they can generate posterior samples rapidly for new observations under the same model and prior assumptions. This amortized structure is particularly useful when repeated inference is required or when simulator evaluations are costly. The advantage becomes more apparent in complex models such as SIRTEM, where data generation is substantially more expensive due to the higher structural and dimensional complexity.

PNPE showed strong predictive performance in several simulation settings, but it incurred the highest computational cost for complex models such as SIRTEM, because it combines a preconditioning step with neural posterior estimation. In addition, unlike fully amortized NPE-based approaches, PNPE may need to be reconditioned or retrained for each new observation, limiting its practicality in settings requiring repeated inference. Overall, these results emphasize that method selection should depend on the intended use case: ABC is attractive for fast single-dataset inference, NPE and NPE-LSTM are advantageous when amortized inference over multiple observations is needed, and PNPE may be useful when improved posterior accuracy justifies the additional computational cost.

### 3.3. Structural and practical identifiability

Our results underscore the distinction between structural and practical identifiability. Structural non-identifiability, as demonstrated in the SEIR and Ebola experiments, led to broad and weakly informative posterior distributions across inference methods. This behavior is consistent with the theoretical expectation that, when the model structure does not allow unique parameter recovery, no inference algorithm can identify a unique parameter value from the available observations alone.

The information gain analysis further supports this interpretation. In structurally identifiable settings, the information gain generally increased as the simulation budget increased, indicating stronger posterior updating relative to the prior. In contrast, under structurally non-identifiable conditions, the information gain remained lower and showed less consistent increases with simulation budget. This suggests that additional simulations alone cannot resolve non-identifiability when multiple parameter combinations produce indistinguishable epidemic trajectories. However, information gain should be interpreted with caution. A larger information gain indicates that the posterior has moved farther from the prior, but it does not necessarily imply accurate or well-calibrated inference, especially if the posterior is overly concentrated.

Practical identifiability, in contrast, depends not only on the model structure but also on the quality and informativeness of the observed data. In our noise-sensitivity experiments, increasing observational noise generally widened predictive intervals and degraded predictive performance, reflecting reduced practical identifiability. Neural SBI methods often achieved lower predictive errors and WIS values than ABC under these noisy conditions, suggesting that they can extract useful information from simulated training data under fixed simulation budgets.

From a theoretical perspective, the behavior of different inference methods reflects their underlying guarantees and limitations. ABC is asymptotically consistent under standard regularity conditions: as the tolerance parameter ε→0 and the number of simulations increase, the ABC posterior converges to the true posterior distribution. This enables calibrated uncertainty estimation in principle, although at substantial computational cost and with known limitations in high-dimensional settings due to the curse of dimensionality. Neural SBI methods, in contrast, rely on learned density approximations and do not provide formal consistency guarantees under finite simulation budgets. Their performance depends on factors such as model capacity, optimization convergence, and the coverage of the observation space by simulated training data. As a result, these methods can be sensitive to simulation–observation mismatch and may produce overly concentrated posterior estimates under certain conditions, leading to overconfident uncertainty quantification.

### 3.4. Summary

Our study demonstrates that SBI provides a powerful and flexible framework for parameter estimation in epidemiological models, particularly when likelihood-based inference is difficult or computationally demanding. Our results show that neural SBI methods can improve predictive accuracy and posterior agreement relative to ABC under constrained simulation budgets, but these gains do not always translate into better uncertainty calibration. In particular, sharper posterior and predictive distributions may lead to undercoverage when uncertainty is underestimated.

The comparison also highlights that no single method is uniformly optimal across all settings. ABC remains useful for simple and fast single-dataset inference, whereas NPE and NPE-LSTM are advantageous when amortized inference is needed across multiple observations. PNPE can improve posterior accuracy in some controlled settings, but its additional preconditioning step increases computational cost and may limit its practicality for repeated inference.

Importantly, our findings emphasize that SBI performance should be interpreted in light of structural and practical identifiability. When parameters are structurally non-identifiable, additional simulations alone cannot resolve the lack of unique parameter recovery. When parameters are structurally identifiable, observational noise and limited data can still reduce practical identifiability and affect posterior uncertainty. Therefore, evaluating SBI methods requires considering not only predictive accuracy, but also uncertainty calibration, computational cost, and identifiability.

This study has several limitations. First, we assume fully observed and complete data without missing observations. In practice, real-world epidemic data often contain missing or delayed observations, and practical identifiability is additionally influenced by the amount, frequency, and type of available data. A range of approaches [25,40,41] has been developed to address this challenge. Second, we do not explicitly consider model misspecification. Amortized simulation-based inference methods rely on the assumption that the simulator is well-specified, i.e., that the observed data are generated from the same model used for simulation. However, in practice, simulators are often idealized representations of complex real-world systems, and this assumption is rarely satisfied [42]. Third, our experiments focused on deterministic models, and extending the framework to stochastic epidemic systems remains an important direction for future work.

Future research should explore hybrid approaches that combine the efficiency of ABC with the accuracy of neural SBI methods, as well as adaptive strategies for handling observational noise. In particular, improving robustness to model misspecification is another critical avenue for future investigation. Extending these methods to real-world outbreak data, incorporating mechanistic constraints or domain-informed priors, and scaling to larger, heterogeneous systems may further improve both the applicability and interpretability of SBI in epidemiological modeling.

## 4. Methods

In this section, we describe the study’s methodology, including the epidemic models considered (Section 4.1), the SBI approaches benchmarked (Section 4.2), and our approach to identifiability (Section 4.3). We then outline the performance metrics (Section 4.4) and the experimental design (Section 4.5) that underpin our comparative evaluation.

### 4.1. Epidemic models

In this study, we consider three epidemic models: SEIR [3], Ebola [2], and SIRTEM [35]. We selected these three models to cover a spectrum of epidemic dynamics, ranging from simple to complex structures. Although deterministic compartmental models such as SEIR are, in principle, amenable to likelihood-based inference, parameter estimation becomes increasingly challenging as model complexity grows. In particular, the presence of latent states and strong parameter correlations can lead to structural and practical non-identifiability, where multiple parameter configurations produce indistinguishable observed trajectories. As a result, posterior distributions may exhibit flat or ridge-like geometries, making reliable inference and uncertainty quantification difficult.

We assume a homogeneous, well-mixed population in which all individuals interact at random with equal probability, regardless of age or immune status. In all models, the epidemic state dynamics are deterministic, and stochasticity is introduced through a probabilistic observation model applied to the model-generated epidemiological quantities.

#### 4.1.1. Model 1: SEIR model.

We first consider the SEIR model, which is a classical compartmental framework that characterizes the dynamics of disease transmission. This model provides a parsimonious baseline for testing the fundamental capacity of SBI methods to recover simple and identifiable dynamics. The compartments are defined as follows: *S* denotes the number of susceptible individuals, *E* the exposed, *I* the infected, and *R* the recovered individuals. The model dynamics are governed by the following system of ordinary differential equations (ODEs):


$$\begin{array}{cccc} & \frac{dS}{dt}=-\beta \frac{IS}{N},\; & S(0) & =S_{0},\\ & \frac{dE}{dt}=\beta \frac{IS}{N}-\kappa E,\; & E(0) & =E_{0},\\ & \frac{dI}{dt}=\kappa E-\gamma I,\; & I(0) & =I_{0},\\ & \frac{dR}{dt}=\gamma I,\; & R(0) & =R_{0}. \end{array}$$

where β is the transmission rate, κ is the rate at which exposed individuals become infectious (1/κ is the latent period), γ is the recovery rate, and *N* is the total population size. A closed population is assumed, such that *N* = *S* + *I* + *E* + *R*. The initial conditions *S*_0_, *E*_0_, *I*_0_, and *R*_0_ correspond to the initial numbers of susceptible, exposed, infected, and recovered individuals, respectively. The simulation generates the newly infected individuals (κE), whose data are used as the observed time-series data for inference.

#### 4.1.2. Model 2: Ebola model.

As the second model, we adopt an Ebola-specific transmission model following [2]. This model incorporates enhanced transmission in healthcare settings. To simplify the analysis, we assume that the transmission arising from deceased individuals is negligible and omit this pathway. The model dynamics are represented by the following system of ODEs:


$$\begin{array}{c} \begin{array}{ccc} \frac{dS}{dt}=-(\beta_{I}I+\beta_{H}H)\frac{S}{N},\; & S(0) & =S_{0},\\ \frac{dE}{dt}=(\beta_{I}I+\beta_{H}H)\frac{S}{N}-\kappa E,\; & E(0) & =E_{0},\\ \frac{dI}{dt}=\kappa E-(\alpha +\gamma_{I}+\delta_{I})I,\; & I(0) & =I_{0},\\ \frac{dH}{dt}=\alpha I-(\gamma_{H}+\delta_{H})H,\; & H(0) & =H_{0},\\ \frac{dR}{dt}=\gamma_{I}I+\gamma_{H}H,\; & R(0) & =R_{0},\\ \frac{dD}{dt}=\delta_{I}I+\delta_{H}H,\; & D(0) & =D_{0}. \end{array} \end{array}$$

The model divides the population into six compartments: *S* the susceptible individuals, *E* the exposed, *I* the infectious, *H* the hospitalized, *R* the recovered, and *D* the dead. New infections occur when susceptible individuals come into contact with either infectious cases or hospitalized cases, with transmission rates $\beta_{I}$ and $\beta_{H}$, respectively. After an average latent period of 1/κ, exposed individuals become infectious. Infectious individuals may be hospitalized at the rate α, recover at the rate $\gamma_{I}$, or die from the disease at the rate $\delta_{I}$. Once hospitalized, individuals either recover at the rate $\gamma_{H}$ or die at the rate $\delta_{H}$. The total population satisfies *N* = *S* + *E* + *I* + *H* + *R* + *D*, and no transmission from deceased individuals is considered in this model. This structure enables explicit examination of nosocomial transmission and disease-outcome heterogeneity, providing a testbed for SBI under multi-pathway infection processes. The simulation generates three distinct epidemiological outputs, which are used as the observed time-series data for inference: (i) the newly infected individuals, given by κE(t); (ii) the new hospitalizations, given by αI(t); and (iii) deaths, given by $\delta_{I}I(t)+\delta_{H}H(t)$.

#### 4.1.3. Model 3: SIRTEM model.

The final model considered is the SIRTEM model introduced by [35,43], representing the most detailed and data-driven scenario, enabling evaluation of SBI scalability to high-dimensional epidemiological systems. This model extends the classical SEIR model by integrating additional mechanisms such as diagnostic testing, quarantine, hospitalization, immunization, and loss of immunity. In contrast to standard compartmental models, SIRTEM provides a highly granular representation of the population by distinguishing subgroups such as: (i) susceptible individuals with no prior infection, (ii) symptomatic and asymptomatic infected individuals, (iii) symptomatic but uninfected individuals, and (iv) individuals who have recovered but are erroneously classified as susceptible due to testing errors.

The transitions between these subgroups are governed by a combination of infection dynamics and testing-driven processes. Key to these processes are the parameters β, ϕ, and *g*. The parameter β represents the number of new daily infections produced by a single infectious individual. The parameter ϕ denotes the daily testing rates applied to different population compartments, allowing the model to differentiate between testin*g* for symptomatic and non-symptomatic individuals. Additionally, the parameter *g*, referred to as the general symptomatic rate, identifies the ratio of susceptible individuals who exhibit COVID-like symptoms, such as fever and cough, due to non-COVID infections like the seasonal flu. This parameter is crucial for explaining the presence of symptomatic individuals who are not COVID-infected but enter the testing pipeline, thereby directly influencing the observed number of negative test results.

The full SIRTEM framework consists of 76 compartments that capture detailed epidemiological states, testing outcomes, and intervention processes. While the original model was formulated as a spatial model for multiple cities or regions, we consider a simplified single-population version that retains the essential mechanisms of testing, isolation, hospitalization, recovery, and loss of immunity. In our experiments, the observed data used for inference consist of four time-series outputs generated by the model: (i) the number of positive test cases, (ii) the number of negative test cases, (iii) hospitalizations, and (iv) deaths. These quantities correspond to the observable surveillance signals produced by the testing, isolation, and disease-progression mechanisms embedded in the SIRTEM framework.

#### 4.1.4. Observation model.

In this study, all epidemic models are formulated with deterministic state-transition dynamics, while stochasticity is introduced through probabilistic observation models to account for measurement error, reporting variability, and unobserved heterogeneity in surveillance data.

For a given parameter vector θ, the epidemic model produces a deterministic model output


$$\begin{array}{c} x(\theta )=\{x_{t}(\theta ){\}}_{t=1}^{T}, \end{array}$$

where $x_{t}(\theta )$ denotes the model-predicted epidemiological quantity at time *t*, such as incidence, hospitalizations, or deaths. The observed *t*ime series used for inference is denoted by


$$\begin{array}{c} y^{obs}=\{y_{t}^{obs}{\}}_{t=1}^{T}. \end{array}$$

Synthetic data are generated by applying a probabilistic observation model to the deterministic model output. Specifically, for a given θ, the simulator generates noisy observations


$$\begin{array}{c} y(\theta )=S(x(\theta )),\;y(\theta )=\{y_{t}{\}}_{t=1}^{T} \end{array}$$

where S(·) denotes the stochastic observation operator. Thus, the underlying epidemic dynamics are deterministic, while randomness enters through the observation process.

**Poisson Noise.** For count data where equidispersion is assumed, we model each observation using a Poisson distribution:


$$\begin{array}{c} y_{t}|\theta \sim Poisson\left(\mu_{t}(\theta )\right), \end{array}$$ (1)

where $\mu_{t}(\theta )$ is the model-predicted mean count at time *t*. Under this model,


$$\begin{array}{c} 𝔼\left[y_{t}|\theta \right]=\mu_{t}(\theta ),\;Var\left(y_{t}|\theta \right)=\mu_{t}(\theta ). \end{array}$$ (2)

**Negative Binomial Noise.** To account for overdispersion in observed count data, we also consider a negative binomial observation model parameterized by its mean $\mu_{t}(\theta )$ and dispersion parameter *r*:


$$\begin{array}{c} y_{t}|\theta \sim NegBin\left(\mu_{t}(\theta ),r\right), \end{array}$$ (3)

where *r* > 0 controls the degree of overdispersion. Under this parameterization,


$$\begin{array}{c} 𝔼\left[y_{t}|\theta \right]=\mu_{t}(\theta ),\;Var\left(y_{t}|\theta \right)=\mu_{t}\left(\theta \right)+\frac{\mu_{t}{\left(\theta \right)}^{2}}{r}. \end{array}$$ (4)

Thus, smaller values of *r* correspond to stronger overdispersion, while larger values of *r* reduce the additional variance. As r→∞, the variance approaches $\mu_{t}(\theta )$, and the negative binomial model converges to the Poisson model.

**Fig 6:**
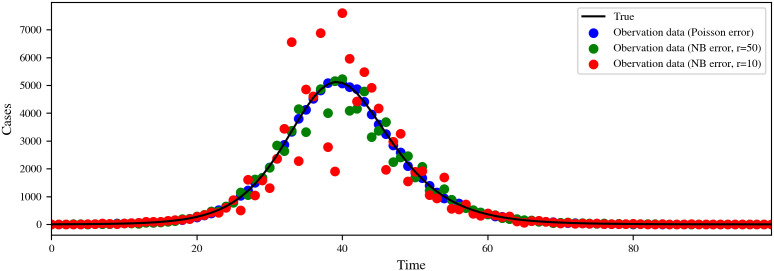
Simulated epidemic trajectory κE under different observation noise models. The black line indicates the true epidemic curve generated from the SEIR model with parameters β=0.80, κ=0.30, and γ=0.34.

Fig 6 displays the SEIR model, along with the observations under different noise models. For the parameter set β=0.80, κ=0.30, and γ=0.34, the Poisson noise model yielded observations that were closely aligned with the true curve. In contrast, negative binomial noise with the dispersion parameter *r* = 50 introduced moderate variability, whereas the *r* = 10 scenario produced substantial overdispersion and outliers. These varying conditions established a systematic framework for assessing the robustness of the inference methods under different levels of observational noise.

### 4.2. Simulation-based inference

SBI refers to the methods for performing Bayesian inference when a likelihood function is unavailable or computationally intractable [12]. In this setting, we consider a simulator that generates synthetic data **y** from parameters θ according to


$$\begin{array}{c} \theta \sim p(\theta ),\;y\sim p(y|\theta ), \end{array}$$

where p(θ) is the prior distribution and p(y∣θ) is implicitly defined by the simulator. Given observed data **y**^*obs*^, the goal of SBI is to approximate the posterior distribution


$$\begin{array}{c} p(\theta |y^{obs})\propto p(y^{obs}|\theta )p(\theta ), \end{array}$$

without requiring explicit evaluation of the likelihood $p(y^{obs}|\theta )$. In this study, we consider several SBI approaches, including ABC, NPE, NPE-LSTM, and PNPE.

#### 4.2.1. Approximate Bayesian Computation (ABC).

ABC is a classical SBI method that directly approximates the posterior distribution $p(\theta |y^{obs})$ [24]. The key idea of ABC is to approximate the posterior distribution by retaining only the parameter values that generate simulated data resembling the observed data. In its simplest form, known as the rejection ABC, the algorithm proceeds as follows: (i) sample parameter values $\theta^{\left(i\right)}$ from the prior p(θ); (ii) generate synthetic data **y**^(*i*)^ from the simulator $p(y|\theta^{\left(i\right)})$; (iii) compute the distance $d(y^{\left(i\right)},y^{obs})$ between the simulated and observed data; and (iv) accept $\theta^{\left(i\right)}$ if $d(y^{\left(i\right)},y^{obs})\leq \varepsilon$, where ε is a predefined tolerance threshold. The accepted samples form an approximate posterior distribution $p_{\varepsilon }(\theta |y^{obs})$ that converges to the true posterior as ε→0:


$$\begin{array}{c} p_{\varepsilon }(\theta |y^{obs})\propto p(\theta )\int 1\left\{d(y,y^{obs})\leq \varepsilon \right\}p(y|\theta )dy, \end{array}$$

where 1(·) is the indicator function.

In practice, the choice of the distance metric d(·,·) and tolerance ε strongly influences the quality of the approximation. Moreover, in high-dimensional settings or when simulations are expensive, the rejection ABC can become computationally prohibitive because very few samples satisfy the tolerance criterion. To address these limitations, SMC-ABC adaptively decreases $\varepsilon_{1}>\varepsilon_{2}>\cdots >\varepsilon_{s}$ to concentrate the computational effort on regions of high posterior density [44,45]. In this study, we employ the SMC-ABC approach to perform parameter inference because of its improved efficiency compared to the basic rejection ABC algorithm.

#### 4.2.2. Neural Posterior Estimation (NPE).

NPE is an SBI approach that uses neural networks as conditional density estimators to directly approximate the posterior distribution $p(\theta |y^{obs})$ from simulated data [32,33]. To parameterize the conditional density, we utilize normalizing flows. Normalizing flows are a class of neural networks that learn to transform a simple base distribution, such as a standard Gaussian, into a complex target distribution through a series of invertible and differentiable mappings. In the context of NPE, they provide the flexibility needed to approximate non-standard, skewed, or multimodal posterior distributions often encountered in epidemiological systems.

Unlike ABC, which relies on accept/reject sampling, NPE trains a conditional density estimator $q_{\phi }(\theta |y)$ using simulated parameter–data pairs $\{(\theta^{\left(i\right)},y^{\left(i\right)}){\}}_{i=1}^{N}$, where


$$\begin{array}{c} \theta^{\left(i\right)}\sim p(\theta ),\;y^{\left(i\right)}\sim p(y|\theta^{\left(i\right)}). \end{array}$$

The density estimator is trained by minimizing the negative log-likelihood loss:


$$\begin{array}{c} ℒ(\phi )=-\sum_{i=1}^{N}\log q_{\phi }(\theta^{\left(i\right)}|{𝐲}^{\left(i\right)}). \end{array}$$

For sufficiently expressive neural networks and density estimators, this procedure learns an approximation to the posterior distribution. Once training is complete, posterior inference for the observed data is obtained by evaluating


$$\begin{array}{c} q_{\phi }\left(\theta |y^{obs}\right)\approx p\left(\theta |y^{obs}\right), \end{array}$$

without requiring additional simulations. The NPE algorithm is described as follows: (i) sample parameter values $\theta^{\left(i\right)}$ from the prior distribution p(θ); (ii) simulate synthetic data **y**^(*i*)^ using the sampled parameters; (iii) collect the pairs $\{(\theta^{\left(i\right)},y^{\left(i\right)}){\}}_{i=1}^{N}$ to form a training dataset; and (iv) train a conditional density estimator to approximate p(θ∣y).

In practice, expressive neural density estimators are used to capture complex, multimodal posterior distributions. In this study, we adopt the Masked Autoregressive Flow (MAF) [46] as our core normalizing flow architecture. This choice is informed by the observation that the posterior distributions of the targeted epidemiological models generally exhibit relatively smooth, unimodal structures. Given these characteristics, MAF provides an optimal balance between parameter efficiency and training stability, avoiding the unnecessary computational overhead associated with more complex spline-based transforms. To ensure the robustness of our model selection, we conducted additional experiments using Neural Spline Flows (NSF) [47]. As shown in Tables N and O in S1 Text, NSF achieved performance comparable to or worse than MAF across the considered evaluation metrics. These results indicate that the posterior distributions in our experiments do not require the additional flexibility provided by NSF and confirm that MAF is sufficiently expressive for the inference tasks considered in this study.

While Sequential NPE (SNPE) offers iterative refinement, we deliberately adopt an amortized NPE approach to ensure computational efficiency across multiple observations. Beyond amortization, our choice is also motivated by concerns regarding information leakage and prior-observation mismatch, which are well-documented challenges in sequential inference frameworks [34,48]. We retain amortized NPE as a baseline and evaluate preconditioning separately in PNPE.

#### 4.2.3. Neural Posterior Estimation with temporal embedding (NPE-LSTM).

While vanilla NPE already performs an implicit embedding of the input data through its feedforward encoder, high-dimensional time-series observations can benefit from more structured representations that explicitly capture temporal dependencies. To this end, we consider a variant of NPE that replaces the standard feedforward encoder with an explicit embedding network.

The embedding network acts as a learnable feature extractor that transforms the high-dimensional simulated data $y\in {\mathbb{R}}^{d}$ into a lower-dimensional latent representation


$$\begin{array}{c} h=E(y;\phi_{E}), \end{array}$$

where $\phi_{E}$ denotes the embedding network parameters. The posterior estimator is then trained conditionally on **h** rather than directly on **y**. In particular, the conditional density estimator becomes


$$\begin{array}{c} q_{\phi }\left(\theta |E(y;\phi_{E})\right)\approx p(\theta |y), \end{array}$$

where the parameters $\phi_{E}$ of the embedding network and the parameters ϕ of the density estimator are optimized jointly. The embedding network can be implemented using convolutional, recurrent, or fully connected architectures, depending on the structure of the simulated data. This NPE with an embedding network approach allows the model to capture relevant summary features of complex data automatically, reducing the effective dimensionality of the conditioning input and improving both the convergence speed and the quality of the inferred posterior.

In this paper, we employ LSTM as an embedding network to address the complexity of the simulated epidemic data.This choice is motivated by the fact that infectious disease dynamics are characterized by strong temporal autocorrelations and lagged effects, where the current observation is intrinsically linked to previous states. Unlike feedforward architectures that treat each time point as an independent feature, LSTMs utilize recurrent gates to preserve the sequential structure and capture the nonlinear dependencies across time, such as the staggered peaks between infections and deaths. Similar recurrent embedding architectures have also been applied in several previous studies on epidemic modeling, demonstrating the effectiveness of sequence-based feature extraction for high-dimensional simulation outputs [19,37].

#### 4.2.4. Preconditioned Neural Posterior Estimation (PNPE).

PNPE is an extension of NPE designed to improve simulation efficiency by guiding neural posterior training toward regions of the parameter space that are most consistent with the observed data [34]. When the prior distribution is broad, standard amortized NPE may expend a large fraction of simulations on parameter values that generate observations far from the target data, resulting in inefficient training.

PNPE mitigates this issue by introducing a lightweight preconditioning step based on ABC. In our implementation, the total simulation budget is split evenly between an ABC-based preconditioning stage and neural posterior estimation. Specifically, 50% of the simulations are allocated to an SMC-ABC procedure to obtain an approximate posterior distribution.

To construct a proposal distribution from the ABC posterior samples, we fit a kernel density estimator (KDE) to the accepted parameter samples. This KDE-based approximation serves as an informed proposal distribution that concentrates the probability mass in regions of the parameter space that are plausible given the observed data.

The remaining 50% of the simulation budget is used to generate the training data for neural posterior estimation by sampling the parameters from this KDE-based proposal distribution and simulating corresponding observations. These parameter-data pairs are subsequently used to train the NPE-LSTM model. By concentrating simulations in regions of high posterior probability, PNPE can improve the efficiency and accuracy of neural posterior estimation. However, because the preconditioning distribution is constructed for a specific observed dataset, PNPE is only partially amortized: posterior sampling is fast after training, but the preconditioning stage may need to be repeated for each new observation. Importantly, this design ensures that PNPE and standard NPE-LSTM are compared under identical total simulation budgets.

#### 4.2.5. Reference Posterior: Bayesian inference.

To evaluate the performance of SBI methods, we require a reliable approximation of the true posterior distribution, which serves as a reference for comparison. We use traditional Bayesian inference to construct a reference posterior. Bayesian inference estimates the posterior distribution $p(\theta |y^{obs})$ using Bayes’ theorem as follows:


$$\begin{array}{c} p(\theta |y^{obs})=\frac{p(y^{obs}|\theta )p(\theta )}{p(y^{obs})}, \end{array}$$

where p(θ) is the prior, $p(y^{obs}|\theta )$ is the likelihood function, and *p*(**y**^*obs*^) is the marginal likelihood. Given a tractable likelihood function, we approximate the posterior using MCMC sampling. Specifically, we employ the No-U-Turn Sampler (NUTS), a variant of the Hamiltonian Monte Carlo (HMC) method, which adaptively tunes the path length to efficiently explore complex, high-dimensional posterior landscapes [49,50]. Bayesian inference under a well-specified likelihood model is considered the gold standard for comparing approximate inference methods. Although not always available in SBI settings due to intractable likelihoods, it provides a valuable benchmark when applicable. This framework is applied to the SEIR and Ebola models to obtain high-quality reference posterior distributions. These serve as benchmarks for assessing the accuracy and calibration of SBI methods such as ABC, NPE, NPE-LSTM, and PNPE.

### 4.3. Identifiability

The concept of identifiability plays a central role in model-based inference and determines whether reliable conclusions can be drawn from observed data. It distinguishes between models that can fit the data meaningfully and those that cannot, regardless of the optimization algorithm or inference method used. Identifiability analysis is essential for constructing models that yield interpretable parameter estimates and well-determined predictions.

#### 4.3.1. Structural identifiability.

A parameter is structurally identifiable if it can be uniquely determined from perfect (noise-free and infinite) observations of the model’s output. More formally, a parameter vector θ is globally structurally identifiable if the following implication holds:


$$\begin{array}{c} y(\theta )=y(\theta^{\prime })\;\Rightarrow \;\theta =\theta^{\prime },\;\forall \theta ,\theta^{\prime }, \end{array}$$

where y(θ) denotes the model output as a function of parameters. Structural identifiability is a theoretical property of the model structure, determined solely by the system of equations and observation functions. It assesses whether model parameters can be uniquely recovered from perfect observations. Importantly, it is independent of any specific dataset and does not rely on actual experimental data, making it a prerequisite for meaningful parameter inference.

Structural identifiability analysis is typically performed using symbolic computational tools that examine model equations and observation functions. Several software packages have been developed for this purpose. In this study, we use the DAISY software [38] to assess the structural identifiability of the two models: the SEIR and Ebola models. Each model is encoded as a system of ordinary differential equations, along with specified observation functions, and analyzed for the global structural identifiability of all parameters. This step ensures that the parameter inference based on these models is theoretically valid, provided sufficient and noise-free data.

#### 4.3.2. Practical identifiability.

Although structural identifiability is a necessary condition, it does not guarantee that parameters can be estimated accurately in practice. Practical identifiability considers the effects of limited, noisy, and potentially uninformative data. A parameter may be structurally identifiable but practically unidentifiable if the available data do not sufficiently constrain it. In such cases, the posterior distribution may remain broad or flat, leading to substantial uncertainty.

Practical identifiability is influenced by multiple factors, including observation noise, the amount and frequency of available data, and the type of observed variables. In this study, we primarily investigate practical identifiability through varying levels of observational noise in order to provide a controlled setting for systematically comparing inference methods across models and identifiability regimes.

Specifically, we simulate noisy observations using Poisson and negative binomial noise models, which are suitable for overdispersed count data commonly encountered in epidemic modeling. By varying the dispersion parameter *r*, we evaluate how inference results change under increasing noise levels. This analysis provides a practical diagnostic of parameter identifiability under realistic observational uncertainty.

### 4.4. Performance metrics

The primary objective of SBI is to recover the posterior distribution. Accordingly, the most principled way to assess performance is to compare the inferred posterior to a reference or ground-truth posterior using a suitable distance measure between the probability distributions [36]. In our study, the posterior predictive check (PPC), Quantitative metrics, maximum mean discrepancy (MMD), and classifier 2-sample test (C2ST) are considered the performance metrics for SBI methods.

#### 4.4.1. Posterior Predictive Check (PPC).

PPC is used to evaluate whether the inferred posterior distribution can generate data consistent with the observed dataset [4]. Given posterior samples $\theta^{\left(i\right)}\sim p(\theta |y^{obs})$, we generate replicated datasets via $y^{\left(m\right)}\sim p(y|\theta^{\left(m\right)})$ and compare the time series patterns of the replicated data $\{y^{\left(m\right)}{\}}_{m=1}^{M}$ with those of the observed data **y**^*obs*^. A well-calibrated posterior model should yield predictive simulations in which the observed data fall within the range of plausible outcomes.

#### 4.4.2. Quantitative metrics.

To quantitatively assess the agreement between the posterior predictive simulations and the observations, we adopt the following four metrics: mean absolute error (MAE), mean squared error (MSE), coverage probability of the 95% predictive intervals, and weighted interval score (WIS).

Let $y_{t}^{obs}$ denote the observed value at time *t* and let ${\hat{y}}_{t}$ denote the posterior predictive mean at time *t* under the estimated parameters. The MAE is defined as:


$$\begin{array}{c} MAE=\frac{1}{T}\sum_{t=1}^{T}\left|{\hat{y}}_{t}-y_{t}^{obs}\right|. \end{array}$$

Similarly, MSE quantifies the average squared deviation between the predicted and observed values:


$$\begin{array}{c} \text{MSE}=\frac{1}{T}\sum_{t=1}^{T}{\left({\hat{y}}_{t}-y_{t}^{obs}\right)}^{2}. \end{array}$$

Let [*L*_*t*_, *U*_*t*_] be the 95% predictive interval bounds of the predicted value at time *t*. The empirical coverage rate is given by:


$$\begin{array}{c} {\text{Coverage}}_{95\%}=\frac{1}{T}\sum_{t=1}^{T}1(L_{t}\leq y_{t}^{obs}\leq U_{t}), \end{array}$$

where 1(·) denotes the indicator function. A well-calibrated posterior should yield coverage close to 0.95.

WIS is a scoring rule designed to evaluate probabilistic forecasts expressed as prediction intervals at various confidence levels. It combines multiple interval scores (IS) into a single metric that reflects both the calibration and the sharpness of the predictive distribution [51,52]. For a given (1−α)×100% central prediction interval with lower and upper bounds $l_{\alpha }$ and $u_{\alpha }$ and an observed value $y^{obs}$, the interval score $IS_{\alpha }(y)$ is defined as:


$$\begin{array}{c} IS_{\alpha }(y^{obs})=(u_{\alpha }-l_{\alpha })+\frac{2}{\alpha }(l_{\alpha }-y^{obs})\cdot 1(y^{obs}<l_{\alpha })+\frac{2}{\alpha }(y^{obs}-u_{\alpha })\cdot 1(y^{obs}>u_{\alpha }), \end{array}$$

where $\left(u_{\alpha }-l_{\alpha }\right)$ measures the width (sharpness) of the interval, $1(y^{obs}<l_{\alpha })$ and $1(y^{obs}>u_{\alpha })$ are indicator functions that impose penalties when *y* falls outside the interval, and the penalty strength scales with $\alpha^{-1}$ to place greater emphasis on narrower intervals. To summarize the entire predictive distribution, WIS averages the interval scores over *K* central predictive intervals with levels $\left(1-\alpha_{1}),\ldots ,(1-\alpha_{K}\right)$, and the absolute error of the predictive median $\hat{y}$. The WIS is then defined as:


$$\begin{array}{c} WIS_{\alpha_{0:K}}(y^{obs})=\frac{1}{K+\frac{1}{2}}\left(w_{0}|\hat{y}-y^{obs}|+\sum_{k=1}^{K}w_{k}\cdot IS_{\alpha_{k}}(y^{obs})\right), \end{array}$$

where $w_{k}=\frac{\alpha_{k}}{2}$ for k=1,…,K and $w_{0}=\frac{1}{2}$. A lower WIS value indicates a predictive distribution that is both sharp and well-calibrated.

#### 4.4.3. Maximum Mean Discrepancy (MMD).

MMD is a kernel-based metric that quantifies the difference between two probability distributions in kernel space. Let *P* and *Q* be two distributions over a domain X, and let k:𝒳×𝒳→ℝ be a positive-definite kernel with associated kernel space H. The mean embeddings of *P* and *Q* in H are defined as


$$\begin{array}{c} \mu_{P}={𝔼}_{x\sim P}[k(x,\cdot )],\;\mu_{Q}={𝔼}_{y\sim Q}[k(y,\cdot )]. \end{array}$$

The kernel space norm then gives the squared MMD for their differences as follows:


$$\begin{array}{c} MMD^{2}(P,Q;H)=||\mu_{p}-\mu_{Q}|{|}_{H}^{2}. \end{array}$$

In this study, we adopt the Gaussian kernel, which is widely used in practice. Under this choice, the MMD is characteristic, meaning that MMD(*P*,*Q*) = 0 holds if and only if *P* = *Q*. A lower MMD value indicates that the distributions are more similar, whereas a higher value reflects greater divergence.

#### 4.4.4. Classifier 2-Sample Test (C2ST).

C2ST is a powerful non-parametric test to quantify the similarity between two distributions based on their samples. The method involves training a classifier to distinguish samples drawn from the inferred posterior and those from a reference posterior. If the two sets are indistinguishable, the classifier’s accuracy will be close to 0.5, indicating that the distributions are similar [53,54]. We use the posterior obtained via traditional Bayesian inference as the reference distribution.

#### 4.4.5. Computational runtime.

To provide a transparent and comprehensive assessment of computational cost, we decompose the total wall-clock runtime of each inference method into three distinct phases: (i) data generation, defined as the time required to produce simulated parameter–data pairs from the epidemic simulator; (ii) training, defined as the time required to fit the density estimator to the simulated data; and (iii) inference, defined as the time required to obtain posterior samples for a given observation.

For NPE and NPE-LSTM, the data generation phase corresponds to forward simulations under parameters sampled from the prior. The training phase involves optimizing the neural density estimator on the simulated dataset, while the inference phase corresponds to a single forward pass through the trained network to obtain posterior samples for a new observation. For ABC, there is no clear separation between data generation and training. Instead, these steps are interleaved within the SMC-ABC procedure, which iteratively simulates parameter–data pairs, evaluates distances, and refines the tolerance schedule across populations. For PNPE, the data generation phase includes the full preconditioning pipeline: running ABC to obtain an approximate posterior, sampling parameter values from the resulting KDE-based proposal distribution, and generating simulated observations from the epidemic simulator. The training phase then involves fitting the neural density estimator on the generated dataset. As with NPE and NPE-LSTM, the inference phase consists of a single forward pass through the trained model. For MCMC, there is no separate training phase. The reported runtime represents the total wall-clock time for running four NUTS chains with 5,000 iterations each, including warm-up and convergence assessment, and retaining 10,000 post-warm-up posterior samples per dataset.

This decomposition highlights an important practical trade-off. Neural methods such as NPE and NPE-LSTM enable near-instantaneous inference once trained but incur substantial upfront training costs. In contrast, classical methods such as MCMC or ABC do not require training but typically involve higher computational costs per inference. Therefore, when only a small number of inference tasks are required, classical methods may be more efficient. Conversely, in settings where inference must be performed repeatedly across many observations, the training cost of neural methods can be amortized, leading to significant computational advantages. Detailed runtime breakdowns by phase are reported in Tables D–F in S1 Text.

#### 4.4.6. Information gain.

To quantify the degree to which each inference method updates the prior toward the posterior, we measure the marginal KL divergence between the inferred posterior and the prior distribution. For a single parameter $\theta_{j}$, the marginal KL divergence is defined as:


$$\begin{array}{c} D_{KL}\left(p(\theta_{j}|y^{obs})\;\|\;p(\theta_{j})\right)=\int p(\theta_{j}|y^{obs})\log\frac{p(\theta_{j}|y^{obs})}{p(\theta_{j})}d\theta_{j}. \end{array}$$

where $p(\theta_{j}|y^{obs})$ is the marginal posterior and $p(\theta_{j})$ is the marginal prior for parameter $\theta_{j}$. A higher value of *D*_KL_ indicates that the posterior has moved substantially away from the prior, reflecting a greater update in the inferred distribution. Conversely, a value near zero suggests that the posterior remains close to the prior, indicating that the prior has not been updated.

In this study, the marginal posterior density is estimated using KDE applied to posterior samples obtained from each SBI method. Let ${\hat{p}}_{j}(\theta_{j}|y^{obs})$ denote the KDE estimate of the marginal posterior density for parameter $\theta_{j}$. We approximate the marginal KL divergence using numerical integration over a grid $\{a_{\ell }{\}}_{\ell =1}^{L}$:


$$\begin{array}{c} {\hat{D}}_{KL,j}=\sum_{\ell =1}^{L}{\hat{p}}_{j}(a_{\ell }|y^{obs})\log\frac{{\hat{p}}_{j}(a_{\ell }|y^{obs})}{p_{j}(a_{\ell })}\Delta a, \end{array}$$

where $p_{j}(a_{\ell })$ is the marginal prior density evaluated at grid point $a_{\ell }$, and Δa is the grid spacing.

We analyze how the marginal KL divergence changes across simulation budgets of 1k, 10k, and 100k. An increase in *D*_KL_ with a growing simulation budget indicates that additional simulations contribute to updating the posterior distribution; however, this does not necessarily imply that the inference is accurate. Conversely, if *D*_KL_ remains unchanged as the simulation budget grows, this suggests that additional simulations fail to further update the posterior, which is characteristic of structurally non-identifiable settings. To benchmark the performance of each SBI method, we use the marginal KL divergence of the MCMC reference posterior as a reference against which the information gain of each SBI method is compared. Note that increases in *D*_*KL*_ should be interpreted with caution in non-identifiable settings, as they may reflect posterior over-concentration rather than a genuine update of the prior.

### 4.5. Experiments

We evaluated the performance of SBI methods across a range of epidemic models and experimental conditions. To simulate realistic inference tasks, we sampled 10 sets of true parameters $\{\theta^{\left(i\right)}{\}}_{i=1}^{10}$ from a prior distribution. For each sampled parameter, we generated a corresponding observation *x*_*i*_ using the epidemic simulator, as shown in Figs 3-5 in S1 Text. Then, we estimated the reference posterior using Bayesian inference, generating 10,000 MCMC samples per dataset using the NUTS sampler.

To evaluate the efficiency of each method, we varied the simulation budget across three levels: 1k, 10k, and 100k simulations. Each SBI method was run independently for each simulation budget, and the resulting posterior estimates were compared to the reference posterior using performance metrics. For the identifiability analysis, we examined its performance under structurally identifiable and non-identifiable parameter configurations. These conditions were determined via structural identifiability analysis using DAISY [38,55]. Specifically, for the SEIR model, we note that, although it is structurally identifiable when initial conditions are known, it becomes non-identifiable when they are unknown. To reflect the uncertainty inherent in the early stages of an actual epidemic, we established a deliberate non-identifiable environment by assuming that the initial conditions, including the initial infected state (*I*_0_), are unknown. For the Ebola model, structural identifiability depends on the availability of observational data streams. When all three components—(i) newly infected individuals, κE(t); (ii) hospitalized individuals, αI(t); and (iii) deaths, $\delta_{I}I(t)+\delta_{H}H(t)$—are observed, the model is structurally identifiable. However, when only (i) and (ii) observations are available, the model becomes structurally non-identifiable. Accordingly, we constructed both identifiable and non-identifiable settings by varying the availability of observational data, which allowed us to systematically evaluate how each method performed under different identifiability conditions.

To evaluate robustness to observational noise, we modeled the data-generating process using a negative binomial distribution with varying dispersion parameters r∈{10,50}. Smaller values of *r* correspond to higher overdispersion, allowing us to assess robustness under increasingly noisy observation regimes. This approach allows us to assess the sensitivity of each inference method to different noise levels in the observations.

#### 4.5.1. Methodological configurations.

To ensure reproducibility and consistency across inference methods, we explicitly documented all hyperparameter settings used in the experiments. Each method was trained once per dataset and simulation budget, using a fixed random seed.

**ABC** We followed the hyperparameter configuration adopted in a recent benchmarking study by Lueckmann et al. (2021) [36]. The target population size was set to 100 for simulation budgets of 1k and 10k, and increased to 1,000 for the 100k budget to ensure a higher resolution of the posterior approximation. The discrepancy between the simulated epidemic trajectories and the observed data was quantified using the Euclidean distance (*L*_2_ norm). For the tolerance schedule, we employed the quantile-epsilon strategy, where the threshold ε for each new population was updated based on the 0.2 quantile of the distance distribution from the preceding population.

**NPE** The training datasets for each model were generated according to the specified simulation budgets of 1k, 10k, and 100k. We adopted the MAF architecture as the conditional density estimator [46], consisting of five transformations and two hidden layers with 50 units each. The neural networks were trained with a learning rate of 0.0005. To optimize training efficiency across different data scales, the batch size was adjusted according to the simulation budget: 64 for 1k, 128 for 10k, and 256 for 100k simulations. To mitigate overfitting, we implemented an early stopping strategy based on a held-out validation set (comprising 10% of the simulation budget), terminating training if the validation log-probability did not improve for 20 consecutive epochs.

**NPE-LSTM** For sequential observations, we used an LSTM-based embedding network to map each epidemic trajectory to a fixed-dimensional summary representation. The embedding network consisted of a single-layer bidirectional LSTM with a hidden dimension of 128. We extracted the last time-step LSTM output, applied dropout with a rate of 0.1, and projected it through a linear layer to obtain a 30-dimensional embedding. This embedding was used as the conditioning input to the neural posterior estimator.

**PNPE** We split the total simulation budget evenly between an ABC-based preconditioning stage and neural posterior estimation. Specifically, 50% of the simulations were allocated to ABC to obtain an approximate posterior, which was used to define a proposal distribution. The remaining 50% of the simulation budget was then used to generate training data for NPE-LSTM by sampling parameters from this proposal distribution. This design ensures that PNPE and standard NPE-LSTM are compared under identical total simulation budgets.

#### 4.5.2. Computational environment.

We utilized publicly available libraries and toolkits to ensure reproducibility and efficiency. Bayesian inference was performed using the BayesianFitForecast toolbox [56], which provides a structured framework for fitting epidemiological models using MCMC-based methods. For ABC, we used the pyABC library [57], which supports sequential and adaptive ABC algorithms with flexible distance metrics and parallel simulation capabilities. For NPE and its variants, we relied on the sbi toolbox [58], a PyTorch-based framework designed for simulation-based inference using neural density estimators such as normalizing flows. For the SIRTEM model, we used the PySIRTEM library [59]. All experiments were executed on the Sol Supercomputer at Arizona State University, using compute nodes with 128 CPU cores (2× AMD EPYC 7713 Zen3 processors) and 512 GiB of RAM [60]. All experiments were implemented in Python 3.11 using PyTorch 2.5.1 and NumPy 2.2.4.

## Supporting information

S1 Text This supporting document contains all supplementary tables and figures cited in the main text. (DOCX)
