# Supplementary material for: A comparative study of simulation-based inference methods for epidemic models with identifiability considerations
Source: PLoS Comput Biol. 2026 Jun 2;22(6):e1014364. doi: 10.1371/journal.pcbi.1014364 (PMC13252848; doi:10.1371/journal.pcbi.1014364)
Supplement: S1 Text — (DOCX) [file pcbi.1014364.s001.docx]

**Table A in S1 Text.** Prior distributions and ground-truth parameter set used in the SEIR model.

|  | **Parameter** | | |
| --- | --- | --- | --- |
|  | $\beta$ | $\kappa$ | $\gamma$ |
| Prior distribution | Uniform(0.01, 1.5) | Uniform(0.01, 0.5) | Uniform(0.01, 0.5) |
| Ground-truth set 1 | 0.80 | 0.30 | 0.34 |
| Ground-truth set 2 | 0.99 | 0.23 | 0.30 |
| Ground-truth set 3 | 0.86 | 0.24 | 0.22 |
| Ground-truth set 4 | 0.80 | 0.33 | 0.30 |
| Ground-truth set 5 | 0.67 | 0.12 | 0.13 |
| Ground-truth set 6 | 0.91 | 0.12 | 0.26 |
| Ground-truth set 7 | 0.68 | 0.11 | 0.14 |
| Ground-truth set 8 | 1.18 | 0.31 | 0.34 |
| Ground-truth set 9 | 1.26 | 0.29 | 0.23 |
| Ground-truth set 10 | 0.62 | 0.32 | 0.20 |

**Table B in S1 Text.** Prior distributions and ground-truth parameter set used in the Ebola model.

|  | **Parameter** | | | | | | | |
| --- | --- | --- | --- | --- | --- | --- | --- | --- |
|  | $\beta_{I}$ | $\beta_{H}$ | $\kappa$ | $\alpha$ | $\gamma_{I}$ | $\delta_{I}$ | $\gamma_{H}$ | $\delta_{H}$ |
| Prior distribution | Uniform  (0.7, 1.3) | Uniform  (0.2, 0.8) | Uniform  (0.1, 0.3) | Uniform  (0.1, 0.3) | Uniform  (0.1, 0.3) | Uniform  (0.001, 0.05) | Uniform  (0.1, 0.4) | Uniform  (0.001, 0.05) |
| Ground-truth set 1 | 1.03 | 0.68 | 0.30 | 0.15 | 0.17 | 0.03 | 0.15 | 0.05 |
| Ground-truth set 2 | 1.13 | 0.52 | 0.26 | 0.25 | 0.19 | 0.02 | 0.13 | 0.02 |
| Ground-truth set 3 | 1.06 | 0.54 | 0.19 | 0.19 | 0.24 | 0.05 | 0.30 | 0.05 |
| Ground-truth set 4 | 1.03 | 0.76 | 0.26 | 0.21 | 0.11 | 0.01 | 0.14 | 0.03 |
| Ground-truth set 5 | 0.95 | 0.24 | 0.12 | 0.10 | 0.23 | 0.01 | 0.16 | 0.04 |
| Ground-truth set 6 | 1.09 | 0.25 | 0.23 | 0.22 | 0.23 | 0.01 | 0.21 | 0.01 |
| Ground-truth set 7 | 0.96 | 0.21 | 0.13 | 0.22 | 0.14 | 0.03 | 0.35 | 0.01 |
| Ground-truth set 8 | 1.24 | 0.70 | 0.29 | 0.22 | 0.13 | 0.01 | 0.13 | 0.01 |
| Ground-truth set 9 | 1.28 | 0.67 | 0.20 | 0.29 | 0.16 | 0.02 | 0.35 | 0.02 |
| Ground-truth set 10 | 0.93 | 0.72 | 0.18 | 0.24 | 0.17 | 0.01 | 0.13 | 0.01 |

**Table C in S1 Text.** Prior distributions and ground-truth parameter set used in the SIRTEM model.

|  | **Parameter** | | |
| --- | --- | --- | --- |
|  | $\beta$ | $\phi_{s}$ | $g$ |
| Prior distribution | Uniform(0.6, 1.2) | Uniform(0.2, 0.6) | Uniform(0.005, 0.01) |
| Ground-truth set 1 | 0.93 | 0.52 | 0.0099 |
| Ground-truth set 2 | 1.03 | 0.41 | 0.0090 |
| Ground-truth set 3 | 0.96 | 0.43 | 0.0073 |
| Ground-truth set 4 | 0.93 | 0.57 | 0.0089 |
| Ground-truth set 5 | 0.85 | 0.23 | 0.0056 |
| Ground-truth set 6 | 0.99 | 0.23 | 0.0082 |
| Ground-truth set 7 | 0.86 | 0.21 | 0.0057 |
| Ground-truth set 8 | 1.14 | 0.53 | 0.0097 |
| Ground-truth set 9 | 1.18 | 0.51 | 0.0076 |
| Ground-truth set 10 | 0.83 | 0.55 | 0.0071 |

**Table D in S1 Text.** Quantitative evaluation of inference methods under the SEIR model with a simulation budget of 100k. Metrics include MSE, MAE, 95% predictive interval (PI) coverage, weighted interval score (WIS), maximum mean discrepancy (MMD), and runtime in seconds.

|  | | **Reference (10k)** | **ABC** | **NPE** | **NPE-LSTM** | **PNPE** |
| --- | --- | --- | --- | --- | --- | --- |
| MSE | | 1,008.32 ±207.68 | 1,044.27 ±212.03 | 1,110.75 ±224.27 | 1,305.41 ±344.93 | 1,011.35 ±206.34 |
| MAE | | 18.31 ±2.07 | 18.81 ±2.27 | 19.71 ±2.30 | 20.92 ±4.09 | 18.30 ±2.06 |
| WIS | | 42.06 ±4.58 | 49.41 ±12.11 | 45.23 ±5.23 | 49.30 ±7.76 | 42.08 ±4.44 |
| 95% PI | | 96.90% ±1.30 | 98.40% ±1.20 | 96.60% ±1.91 | 98.10% ±1.37 | 96.90% ±1.30 |
| MMD | | - | 0.20 ±0.06 | 0.10 ±0.06 | 0.12 ±0.07 | 0.01 ±0.02 |
| C2ST | | - | 0.84 ±0.05 | 0.75 ±0.09 | 0.81 ±0.05 | 0.55 ±0.03 |
| Runtime | Data generation | - | - | 115.66 | 115.66 | 78.40 ±1.96 |
|  | Training | - | - | 1,332.31 | 1,354.47 | 605.22 ±191.17 |
|  | Inference | 514.98 ±105.20 | 31.08 ±7.19 | 0.16 ±0.04 | 0.29 ±0.35 | 0.08 ±0.01 |
|  | Total | 514.98 ±105.20 | 31.08 ±7.19 | 1,448.13 ±0.04 | 1,470.42 ±0.35 | 683.98 ±190.77 |

**Table E in S1 Text.** Quantitative evaluation of inference methods under the Ebola model with a simulation budget of 100k. Metrics include MSE, MAE, 95% predictive interval (PI) coverage, weighted interval score (WIS), maximum mean discrepancy (MMD), and runtime in seconds.

|  | | **Reference (10k)** | **ABC** | **NPE** | **NPE-LSTM** | **PNPE** |
| --- | --- | --- | --- | --- | --- | --- |
| MSE | Case1 | 894.97 ±180.19 | 1,378.12 ±493.50 | 1,303.89 ±832.28 | 1,377.10 ±640.22 | 901.94  ±175.98 |
|  | Case2 | 460.65 ±210.24 | 644.70 ±353.91 | 568.40 ±383.46 | 558.23 ±281.70 | 468.31  ±213.13 |
|  | Case3 | 107.90 ±72.31 | 129.45 ±75.94 | 124.65 ±82.68 | 113.06 ±71.72 | 108.08  ±72.68 |
| MAE | Case1 | 16.66  ±2.23 | 20.22  ±4.78 | 18.80  ±3.35 | 20.11  ±3.78 | 16.72  ±2.08 |
|  | Case2 | 12.1  ±1.45 | 13.56  ±2.11 | 12.90  ±2.22 | 13.04  ±1.80 | 12.14  ±1.49 |
|  | Case3 | 5.73  ±1.90 | 6.28  ±1.95 | 6.19  ±2.03 | 5.79  ±1.92 | 5.73  ±1.95 |
| WIS | Case1 | 39.66  ±5.21 | 61.42  ±18.58 | 45.37  ±6.63 | 50.68  ±5.20 | 39.77  ±5.01 |
|  | Case2 | 28.33  ±3.28 | 41.02  ±6.75 | 30.73  ±4.91 | 31.77  ±4.99 | 28.58  ±3.29 |
|  | Case3 | 13.39  ±4.36 | 16.02  ±4.36 | 14.56  ±4.66 | 13.80  ±4.25 | 13.42  ±4.34 |
| 95% PI | Case1 | 96.20%  ±1.87 | 98.90%  ±0.99 | 97.70%  ±1.49 | 98.00% ±1.56 | 96.30%  ±1.42 |
|  | Case2 | 95.80%  ±1.93 | 98.50%  ±1.18 | 96.50%  ±1.58 | 97.10% ±1.60 | 96.00%  ±1.56 |
|  | Case3 | 97.20%  ±0.92 | 98.80%  ±0.79 | 97.20%  ±1.23 | 97.80% ±0.92 | 97.30%  ±0.82 |
| MMD | | - | 0.37 ±0.08 | 0.29 ±0.12 | 0.20 ±0.11 | 0.15 ±0.09 |
| C2ST | | - | 0.99 ±0.00 | 0.93 ±0.04 | 0.89  ±0.05 | 0.81 ±0.05 |
| Runtime | Data generation | - | - | 201.15 | 201.15 | 127.99 ±4.45 |
|  | Training | - | - | 2,731.63 | 2,144.99 | 2,296.14 ±713.50 |
|  | Inference | 3,339.46 ±764.53 | 54.17 ±17.02 | 0.18 ±0.03 | 0.22 ±0.20 | 0.13 ±0.02 |
|  | Total | 3,339.46 ±764.53 | 54.17  ±17.02 | 2,932.78 ±0.03 | 2,346.14 ±0.20 | 2,323.57  ±715.84 |

**Table F in S1 Text.** Quantitative evaluation of inference methods under the SIRTEM model with a simulation budget of 10k. Metrics include MSE, MAE, weighted interval score (WIS), and runtime in seconds.

|  | | **ABC** | **NPE** | **NPE-LSTM** | **PNPE** |
| --- | --- | --- | --- | --- | --- |
| MSE | Case1 | 36,339.79  ±7264.37 | 129,424.37  ±122,742.89 | 106,305.93  ±71,478.18 | 37,189.20  ±9,770.28 |
|  | Case2 | 33,172.77  ±11,439.94 | 48,544.09  ±19,479.38 | 47,174.92  ±21,059.55 | 34,115.40  ±11,789.62 |
|  | Case3 | 150,431.81  ±44,806.15 | 206,042.17  ±107,772.34 | 213,766.23  ±63,046.84 | 149,539.89  ±46,276.51 |
|  | Case4 | 30,184.25  ±4,956.70 | 32,784.87  ±7,019.08 | 33,774.50  ±5,582.14 | 30,145.70  ±4,840.86 |
| MAE | Case1 | 109.67  ±11.87 | 181.20  ±87.34 | 173.35  ±56.83 | 108.61  ±13.59 |
|  | Case2 | 128.22  ±25.31 | 152.06  ±31.36 | 145.44  ±35.65 | 128.76  ±25.18 |
|  | Case3 | 222.16  ±39.52 | 262.74  ±81.20 | 273.77  ±47.77 | 221.25  ±41.14 |
|  | Case4 | 98.23  ±9.83 | 104.23  ±14.98 | 105.33  ±11.93 | 98.18  ±9.65 |
| WIS | Case1 | 332.79  ±33.13 | 485.05  ±171.49 | 454.76  ±91.93 | 548.65  ±74.97 |
|  | Case2 | 417.48  ±85.26 | 401.74  ±101.51 | 405.31  ±100.00 | 673.37  ±139.54 |
|  | Case3 | 1,034.83  ±235.16 | 921.60  ±323.12 | 936.68  ±241.16 | 1,242.50  ±241.77 |
|  | Case4 | 509.42  ±63.52 | 422.41  ±83.37 | 422.79  ±81.91 | 565.99  ±56.40 |
| 95% PI | Case1 | 46.30% ±5.01 | 70.50% ±9.61 | 77.30% ±10.80 | 18.20% ±4.64 |
|  | Case2 | 61.00% ±9.84 | 86.00% ±10.57 | 94.00% ±7.38 | 20.70% ±3.50 |
|  | Case3 | 22.30% ±5.17 | 49.20% ±5.14 | 55.60% ±9.62 | 10.00% ±2.29 |
|  | Case4 | 14.00% ±3.68 | 37.80% ±6.05 | 46.00% ±8.72 | 4.50% ±1.65 |
| Runtime | Data generation | - | 3,868.57 | 3,868.57 | 6,190.38 ±263.82 |
|  | Training | - | 661.30 | 3,010.00 | 1,983.10 ±457.26 |
|  | Inference | 4,913.91  ±273.15 | 0.13 ±0.07 | 0.08 ±0.03 | 0.06 ±0.01 |
|  | Total | 4,913.91  ±273.15 | 4,530.10 ±0.07 | 6,878.66 ±0.03 | 8,173.49  ±577.36 |

**Table G in S1 Text.** Simulation time (in seconds) across different models (SEIR, Ebola, and SIRTEM) for varying simulation budgets (1k, 10k, and 100k).

|  | SEIR model | Ebola model | SIRTEM model |
| --- | --- | --- | --- |
| 1k | 1.16 | 1.98 | 43.51 |
| 10k | 11.53 | 20.01 | 383.83 |
| 100k | 115.66 | 201.15 | 3,868.57 |

**Table H in S1 Text.** Structural identifiability regimes are examined for the SEIR and Ebola models under various observation settings and initial condition assumptions.

| **Model** | **Observation data** | **Initial condition** | **Identifiable parameters** |
| --- | --- | --- | --- |
| SEIR | Newly infected individuals | Initial conditions (S, E, I, R) are known | All parameters structurally identifiable |
|  | Newly infected individuals | Initial conditions (S, E, I, R) are unknown | Transmission rate ($\beta$), Total population number ($N$) are structurally non-identifiable |
| Ebola | Newly infected individuals, Hospitalized individuals,  Deaths | Initial conditions (S, E, H, I, R, D) are unknown | All parameters structurally identifiable |
|  | Newly infected individuals, Hospitalized individuals | Initial conditions (S, E, H, I, R, D) are unknown | Hospitalization- and death-related transmission and transition parameters become structurally non-identifiable. Specifically, $\beta_{H}$, $\beta_{I}$, $\delta_{H}$, $\delta_{I}$, $\gamma_{H}$, $\gamma_{I}$ are structurally non-identifiable. |

**Table I in S1 Text.** Information gain for parameter estimation performance across simulation budgets (1k, 10k, and 100k) and identifiability scenarios in the SEIR model.

|  | **ABC** | | | **NPE** | | | **NPE-LSTM** | | | **PNPE** | | |
| --- | --- | --- | --- | --- | --- | --- | --- | --- | --- | --- | --- | --- |
|  | 1k | 10k | 100k | 1k | 10k | 100k | 1k | 10k | 100k | 1k | 10k | 100k |
|  | Structurally identifiable | | | | | | | | | | | |
| $\beta$ | 0.30 ±0.22 | 1.91 ±0.42 | 1.97 ±0.36 | 0.53 ±0.15 | 0.52 ±0.22 | 2.34 ±0.32 | 0.32 ±0.12 | 1.04 ±0.33 | 2.36 ±0.28 | 0.37 ±0.12 | 2.55 ±0.61 | 2.91 ±0.28 |
| $\kappa$ | 0.15 ±0.10 | 1.68 ±0.77 | 1.77 ±0.74 | 0.54 ±0.11 | 0.56 ±0.15 | 2.38 ±0.77 | 0.19 ±0.08 | 0.35 ±0.06 | 2.34 ±0.73 | 0.30 ±0.19 | 2.56 ±1.02 | 2.93 ±0.72 |
| $\gamma$ | 0.08 ±0.04 | 1.56 ±0.47 | 1.58 ±0.42 | 0.38 ±0.13 | 0.52 ±0.16 | 1.86 ±0.37 | 0.05 ±0.02 | 0.96 ±0.30 | 1.93 ±0.26 | 0.12 ±0.10 | 2.18 ±0.36 | 2.43 ±0.32 |
|  | Structurally non-identifiable | | | | | | | | | | | |
| $\beta$ | 0.18 ±0.08 | 0.53 ±0.30 | 0.41 ±0.19 | 0.40 ±0.08 | 0.37 ±0.16 | 0.74 ±0.15 | 0.08 ±0.00 | 0.23 ±0.13 | 0.63 ±0.24 | 0.26 ±0.15 | 0.93 ±0.38 | 0.93 ±0.42 |
| $\kappa$ | 0.16 ±0.09 | 0.91 ±0.30 | 0.50 ±0.29 | 0.42 ±0.05 | 0.63 ±0.17 | 0.96 ±0.39 | 0.09 ±0.00 | 0.39 ±0.10 | 0.95 ±0.57 | 0.51 ±0.25 | 1.58 ±0.61 | 1.48 ±0.63 |
| $\gamma$ | 0.07 ±0.04 | 0.12 ±0.10 | 0.06 ±0.04 | 0.35 ±0.08 | 0.37 ±0.15 | 0.14 ±0.05 | 0.09 ±0.00 | 0.17 ±0.10 | 0.10 ±0.09 | 0.14 ±0.09 | 0.38 ±0.24 | 0.30 ±0.26 |

**Table J in S1 Text.** Information gain for parameter estimation performance across simulation budgets and identifiability scenarios in the Ebola model.

|  | **ABC** | | **NPE** | | | | **NPE-LSTM** | | | **PNPE** | | |
| --- | --- | --- | --- | --- | --- | --- | --- | --- | --- | --- | --- | --- |
|  | 1k | 10k |  | 1k | 10k | 100k | 1k | 10k | 100k | 1k | 10k |  |
| Structurally identifiable | | | | | | | | | | | | |
| $\beta_{I}$ | 0.39 ±0.25 | 1.65 ±1.15 |  | 0.49 ±0.20 | 0.64 ±0.53 | 0.80 ±0.54 | 0.11 ±0.01 | 0.57 ±0.35 | 1.78 ±0.03 | 0.63 ±0.51 | 1.67 ±0.93 |  |
| $\beta_{H}$ | 0.13 ±0.12 | 0.92 ±0.92 |  | 0.14 ±0.03 | 0.17 ±0.08 | 0.26 ±0.24 | 0.11 ±0.00 | 0.15 ±0.07 | 0.60 ±0.02 | 0.15 ±0.11 | 0.86 ±0.57 |  |
| $\kappa$ | 0.37 ±0.23 | 1.86 ±0.74 |  | 0.91 ±0.44 | 0.99 ±0.35 | 1.39 ±0.42 | 0.08 ±0.00 | 0.99 ±0.27 | 2.63 ±0.52 | 0.57 ±0.38 | 2.06 ±0.90 |  |
| $\alpha$ | 0.20 ±0.22 | 1.62 ±0.69 |  | 0.90 ±0.22 | 0.92 ±0.28 | 1.28 ±0.33 | 0.10 ±0.00 | 0.86 ±0.29 | 2.40 ±0.48 | 0.34 ±0.19 | 1.70 ±0.35 |  |
| $\gamma_{I}$ | 0.28 ±0.23 | 1.67 ±1.09 |  | 0.27 ±0.09 | 0.81 ±0.48 | 1.00 ±0.41 | 0.09 ±0.00 | 0.71 ±0.24 | 1.41 ±0.03 | 0.51 ±0.45 | 1.67 ±1.00 |  |
| $\gamma_{H}$ | 0.09 ±0.06 | 0.97 ±1.11 |  | 0.11 ±0.03 | 0.32 ±0.28 | 0.72 ±0.56 | 0.10 ±0.00 | 0.22 ±0.17 | 0.30 ±0.03 | 0.16 ±0.17 | 1.04 ±0.79 |  |
| $\delta_{I}$ | 0.25 ±0.19 | 1.39 ±0.87 |  | 0.71 ±0.29 | 0.90 ±0.22 | 0.93 ±0.26 | 0.10 ±0.00 | 0.83 ±0.25 | 1.62 ±0.09 | 0.50 ±0.43 | 1.54 ±0.64 |  |
| $\delta_{H}$ | 0.15 ±0.14 | 1.06 ±1.04 |  | 0.12 ±0.05 | 0.26 ±0.15 | 0.29 ±0.23 | 0.09 ±0.01 | 0.20 ±0.08 | 0.69 ±0.21 | 0.16 ±0.12 | 0.98 ±0.67 |  |
| Structurally non-identifiable | | | | | | | | | | | | |
| $\beta_{I}$ | 0.22 ±0.15 | 0.79 ±0.36 |  | 0.41 ±0.38 | 0.54 ±0.35 | 0.55 ±0.35 | 0.36 ±0.12 | 0.47 ±0.28 | 0.48 ±0.01 | 0.32 ±0.18 | 1.03 ±0.45 |  |
| $\beta_{H}$ | 0.08 ±0.05 | 0.20 ±0.27 |  | 0.08 ±0.02 | 0.15 ±0.06 | 0.14 ±0.08 | 0.14 ±0.04 | 0.13 ±0.04 | 0.12 ±0.00 | 0.10 ±0.04 | 0.70 ±0.68 |  |
| $\kappa$ | 0.25 ±0.17 | 0.96 ±0.50 |  | 0.32 ±0.26 | 0.51 ±0.26 | 0.48 ±0.20 | 0.33 ±0.20 | 0.43 ±0.23 | 0.51 ±0.01 | 0.34 ±0.28 | 1.31 ±0.52 |  |
| $\alpha$ | 0.07 ±0.04 | 0.95 ±0.58 |  | 0.19 ±0.07 | 0.78 ±0.33 | 0.79 ±0.23 | 0.10 ±0.01 | 0.58 ±0.17 | 0.50 ±0.03 | 0.12 ±0.14 | 1.43 ±0.32 |  |
| $\gamma_{I}$ | 0.08 ±0.05 | 0.55 ±0.64 |  | 0.16 ±0.10 | 0.48 ±0.34 | 0.43 ±0.39 | 0.23 ±0.14 | 0.32 ±0.27 | 0.33 ±0.01 | 0.18 ±0.11 | 0.67 ±0.45 |  |
| $\gamma_{H}$ | 0.06 ±0.02 | 0.38 ±0.35 |  | 0.09 ±0.03 | 0.11 ±0.02 | 0.12 ±0.02 | 0.12 ±0.01 | 0.12 ±0.02 | 0.10 ±0.00 | 0.08 ±0.03 | 0.46 ±0.44 |  |
| $\delta_{I}$ | 0.14 ±0.12 | 0.57 ±0.72 |  | 0.23 ±0.24 | 0.39 ±0.30 | 0.46 ±0.49 | 0.16 ±0.06 | 0.30 ±0.20 | 0.34 ±0.01 | 0.17 ±0.09 | 0.61 ±0.42 |  |
| $\delta_{H}$ | 0.05 ±0.02 | 0.22 ±0.14 |  | 0.12 ±0.07 | 0.13 ±0.05 | 0.14 ±0.06 | 0.11 ±0.02 | 0.11 ±0.01 | 0.11 ±0.00 | 0.09 ±0.03 | 0.55 ±0.50 |  |

**Table K in S1 Text.** Quantitative evaluation of inference methods under the SEIR model with a simulation budget of 100k. Metrics include MSE, MAE, and weighted interval score (WIS).

|  | **Condition** | **ABC** | **NPE** | **NPE-LSTM** | **PNPE** |
| --- | --- | --- | --- | --- | --- |
| MSE | Poisson noise | 1,044.27 ±212.03 | 1,110.75 ±224.27 | 1,305.41 ±344.93 | 1,011.35 ±206.34 |
|  | Negative binomial noise (r=50) | 32,475.23  ±29,537.98 | 3,181.79  ±1,424.26 | 4,469.61  ±1,628.43 | 2,949.38  ±1,381.18 |
|  | Negative binomial noise (r=10) | 47,407.46 ±33,444.89 | 10,804.57  ±16,523.04 | 5,197.02  ±3,507.85 | 6,442.30  ±5,226.45 |
| MAE | Poisson noise | 18.81  ±2.27 | 19.71  ±2.30 | 20.92  ±4.09 | 18.30  ±2.06 |
|  | Negative binomial noise (r=50) | 92.63  ±50.96 | 30.35  ±6.10 | 34.67  ±5.72 | 28.30  ±5.16 |
|  | Negative binomial noise (r=10) | 118.89  ±45.41 | 47.31  ±26.38 | 37.96  ±10.40 | 41.02  ±14.17 |
| WIS | Poisson noise | 49.41  ±12.11 | 45.23  ±5.23 | 49.30  ±7.76 | 42.08  ±4.44 |
|  | Negative binomial noise (r=50) | 293.94  ±134.53 | 75.01  ±11.26 | 79.87  ±10.90 | 63.83  ±10.55 |
|  | Negative binomial noise (r=10) | 368.35  ±124.34 | 87.11  ±56.72 | 94.76  ±13.32 | 93.82  ±25.47 |

**Table L in S1 Text.** Quantitative evaluation of inference methods under the Ebola model with a simulation budget of 100k. Metrics include MSE, MAE, and weighted interval score (WIS).

|  |  | **Condition** | **ABC** | **NPE** | **NPE-LSTM** | **PNPE** |
| --- | --- | --- | --- | --- | --- | --- |
| MSE | Case1 | Negative binomial noise (r=50) | 2,863.53  ±1,430.23 | 2,105.85  ±2,369.15 | 1,656.96  ±757.81 | 1,640.28  ±752.22 |
|  |  | Negative binomial noise (r=10) | 4,782.95  ±3,981.36 | 3,252.53  ±3,088.35 | 3,396.58  ±2,621.58 | 2,621.24  ±2,203.07 |
|  | Case2 | Negative binomial noise (r=50) | 3,038.39  ±2,644.15 | 802.91  ±309.97 | 802.36  ±420.99 | 697.18  ±267.38 |
|  |  | Negative binomial noise (r=10) | 7,309.44  ±10,625.38 | 2,671.81  ±2,642.64 | 3,136.51  ±301.85 | 4,521.18  ±6,769.50 |
|  | Case3 | Negative binomial noise (r=50) | 183.56  ±137.46 | 146.07  ±84.08 | 149.37  ±92.69 | 140.84  ±86.06 |
|  |  | Negative binomial noise (r=10) | 328.37  ±279.50 | 287.34  ±228.70 | 301.85  ±284.28 | 212.56  ±146.16 |
| MAE | Case1 | Negative binomial noise (r=50) | 28.58  ±10.54 | 23.19  ±9.29 | 21.62  ±4.20 | 21.77  ±4.96 |
|  |  | Negative binomial noise (r=10) | 36.06  ±17.89 | 27.94  ±10.08 | 29.38  ±8.76 | 25.86  ±8.25 |
|  | Case2 | Negative binomial noise (r=50) | 26.22  ±10.71 | 15.47  ±2.84 | 15.45  ±2.66 | 14.96  ±2.71 |
|  |  | Negative binomial noise (r=10) | 33.89  ±23.45 | 24.50  ±11.04 | 25.16  ±8.76 | 27.32  ±18.16 |
|  | Case3 | Negative binomial noise (r=50) | 7.13  ±2.51 | 6.62  ±2.05 | 6.63  ±2.26 | 6.41  ±2.21 |
|  |  | Negative binomial noise (r=10) | 9.29  ±4.34 | 8.73  ±3.45 | 8.82  ±4.01 | 7.74  ±2.75 |
| WIS | Case1 | Negative binomial noise (r=50) | 101.61  ±38.78 | 56.03  ±20.44 | 52.88  ±6.07 | 50.31  ±9.47 |
|  |  | Negative binomial noise (r=10) | 118.49  ±43.40 | 67.26  ±14.66 | 69.09  ±11.68 | 62.06  ±13.39 |
|  | Case2 | Negative binomial noise (r=50) | 74.35  ±22.29 | 37.54  ±5.77 | 38.15  ±5.36 | 35.42  ±5.81 |
|  |  | Negative binomial noise (r=10) | 92.60  ±39.27 | 57.49  ±23.43 | 56.91  ±20.41 | 66.53  ±13.39 |
|  | Case3 | Negative binomial noise (r=50) | 20.10  ±6.25 | 15.51  ±4.28 | 15.47  ±5.08 | 14.93  ±4.87 |
|  |  | Negative binomial noise (r=10) | 24.23  ±8.89 | 20.48  ±7.25 | 21.02  ±9.16 | 18.23  ±6.48 |

**Table M in S1 Text.** Quantitative evaluation of inference methods with a simulation budget of 10k. Metrics include MSE, MAE, 95% predictive interval (PI) coverage, and weighted interval score (WIS).

|  | **ABC** | **NPE** | **NPE-LSTM** | **PNPE** |
| --- | --- | --- | --- | --- |
| MSE | 21,819.61 | 17,841.27 | 13,719.96 | 21,621.76 |
| MAE | 65.51 | 64.10 | 67.16 | 66.81 |
| WIS | 199.11 | 238.51 | 152.85 | 163.45 |
| 95% PI | 100.00% | 96.67% | 86.67% | 86.67% |

**Table N in S1 Text.** Comparison of MAF and NSF performance for parameter estimation in the SEIR model.

|  |  | **NPE** | | **NPE-LSTM** | |
| --- | --- | --- | --- | --- | --- |
|  |  | **MAF** | **NSF** | **MAF** | **NSF** |
| MMD |  | 0.10 ±0.06 | 0.12 ±0.09 | 0.12 ±0.07 | 0.29 ±0.05 |
| C2ST |  | 0.75 ±0.09 | 0.71 ±0.07 | 0.82 ±0.05 | 0.91 ±0.02 |
| MSE |  | 1,110.75 ±224.27 | 1,081.08 ±219.89 | 1,305.41 ±344.93 | 1,425.10 ±509.37 |
| MAE |  | 19.71 ±2.27 | 18.94 ±1.90 | 20.92 ±4.09 | 21.75 ±3.37 |

**Table O in S1 Text.** Comparison of MAF and NSF performance for parameter estimation in the Ebola model.

|  |  | **NPE** | | **NPE-LSTM** | |
| --- | --- | --- | --- | --- | --- |
|  |  | **MAF** | **NSF** | **MAF** | **NSF** |
| MMD | | 0.29 ±0.12 | 0.36 ±0.14 | 0.20 ±0.11 | 0.59 ±0.05 |
| C2ST | | 0.93 ±0.04 | 0.96 ±0.03 | 0.89 ±0.05 | 1.00 |
| MSE | Case1 | 1,303.89 ±832.28 | 1,647.07 ±956.26 | 1,377.10 ±640.22 | 2,028,260.45 ±1,643,588.95 |
|  | Case2 | 568.40 ±383.46 | 569.34 ±265.35 | 558.23 ±281.70 | 871,170.20 ±543,654.37 |
|  | Case3 | 124.65 ±82.68 | 129.15 ±70.90 | 113.06 ±71.72 | 31,368.00 ±34,109.48 |
| MAE | Case1 | 18.80 ±3.35 | 22.12 ±8.82 | 20.11 ±3.78 | 834.42 ±344.35 |
|  | Case2 | 12.90 ±2.22 | 13.49 ±3.35 | 13.04 ±1.80 | 484.49 ±120.91 |
|  | Case3 | 6.19 ±2.03 | 6.29 ±1.84 | 5.79 ±1.92 | 87.85 ±48.76 |

**
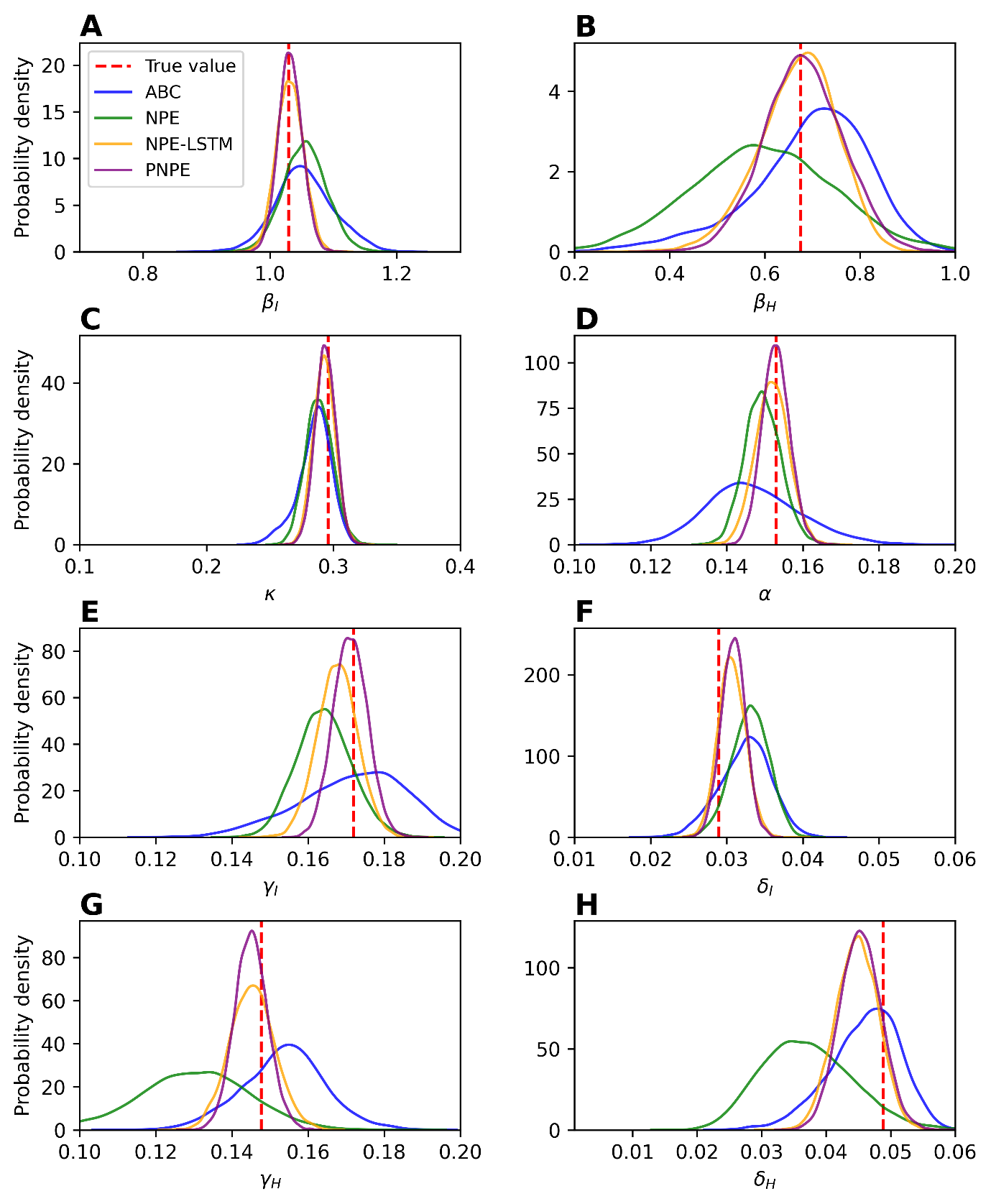
**

**Fig 1 in S1 Text. Posterior distributions of parameters of the Ebola model. A** $\beta_{I}$, **B** $\beta_{H}$, **C** $\kappa$, **D** $\alpha$, **E** $\gamma_{I}$, **F** $\delta_{I}$, **G** $\gamma_{H}$, and **H** $\delta_{H}$ are estimated by ABC, NPE, NPE-LSTM, and PNPE. The red dashed line indicates the true parameter value.

**
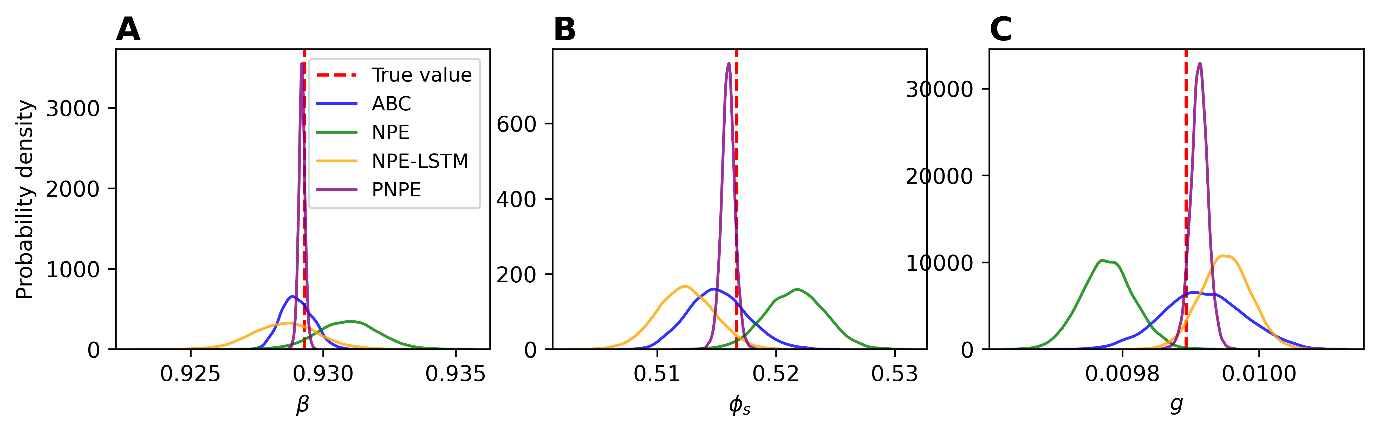
Fig 2 in S1 Text. Posterior distributions of parameters of the SIRTEM model. A** $\beta$, **B** $\phi_{s}$, and **C** $g$ are estimated by ABC, NPE, NPE-LSTM, and PNPE. The red dashed line indicates the true parameter value.

**
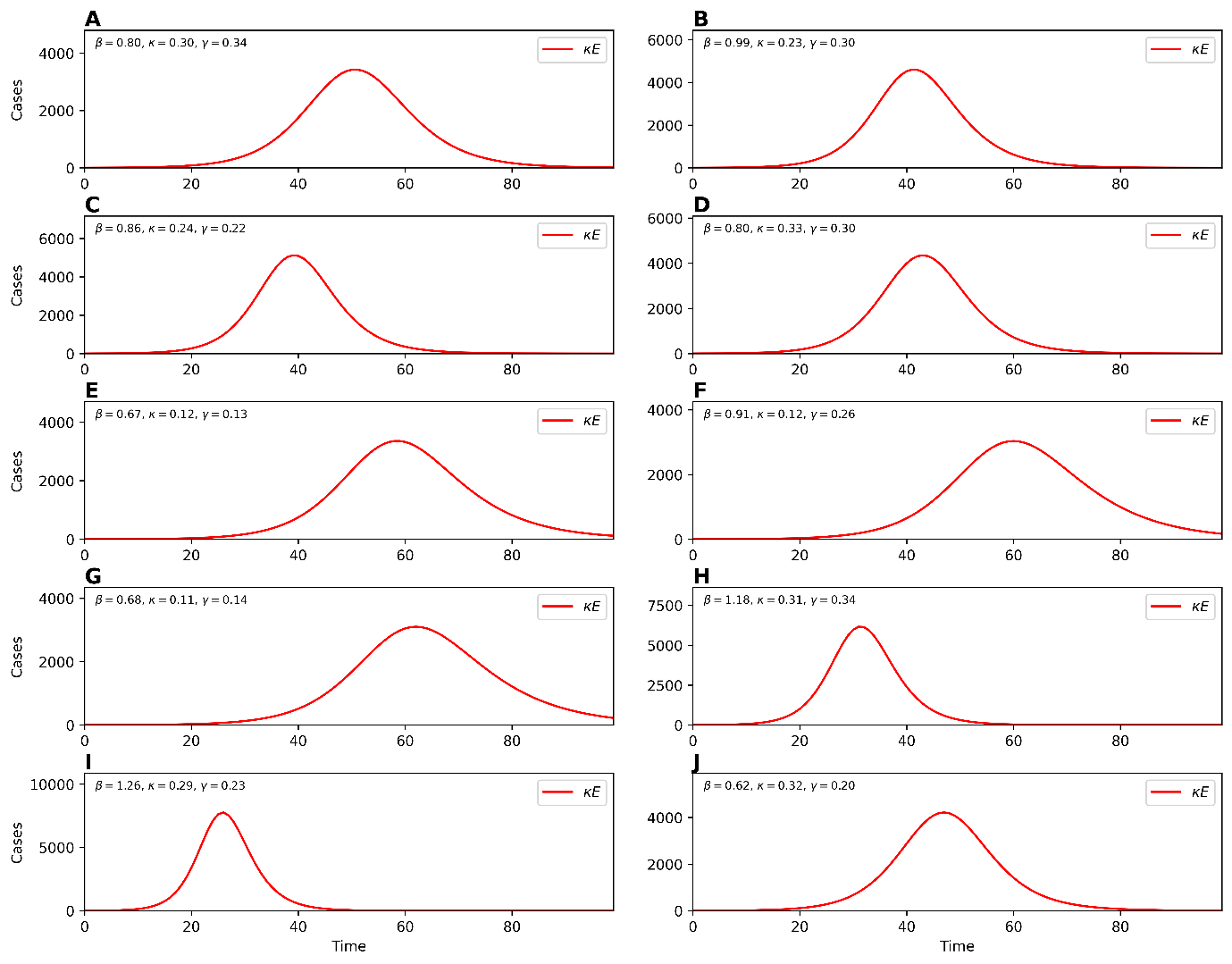
Fig 3 in S1 Text. Synthetic epidemic trajectories generated from the SEIR model using 10 distinct parameter sets.** **A–J** display one simulated trajectory with its corresponding parameter values ($\beta$, $\kappa$, and $\gamma$) indicated in the legend.


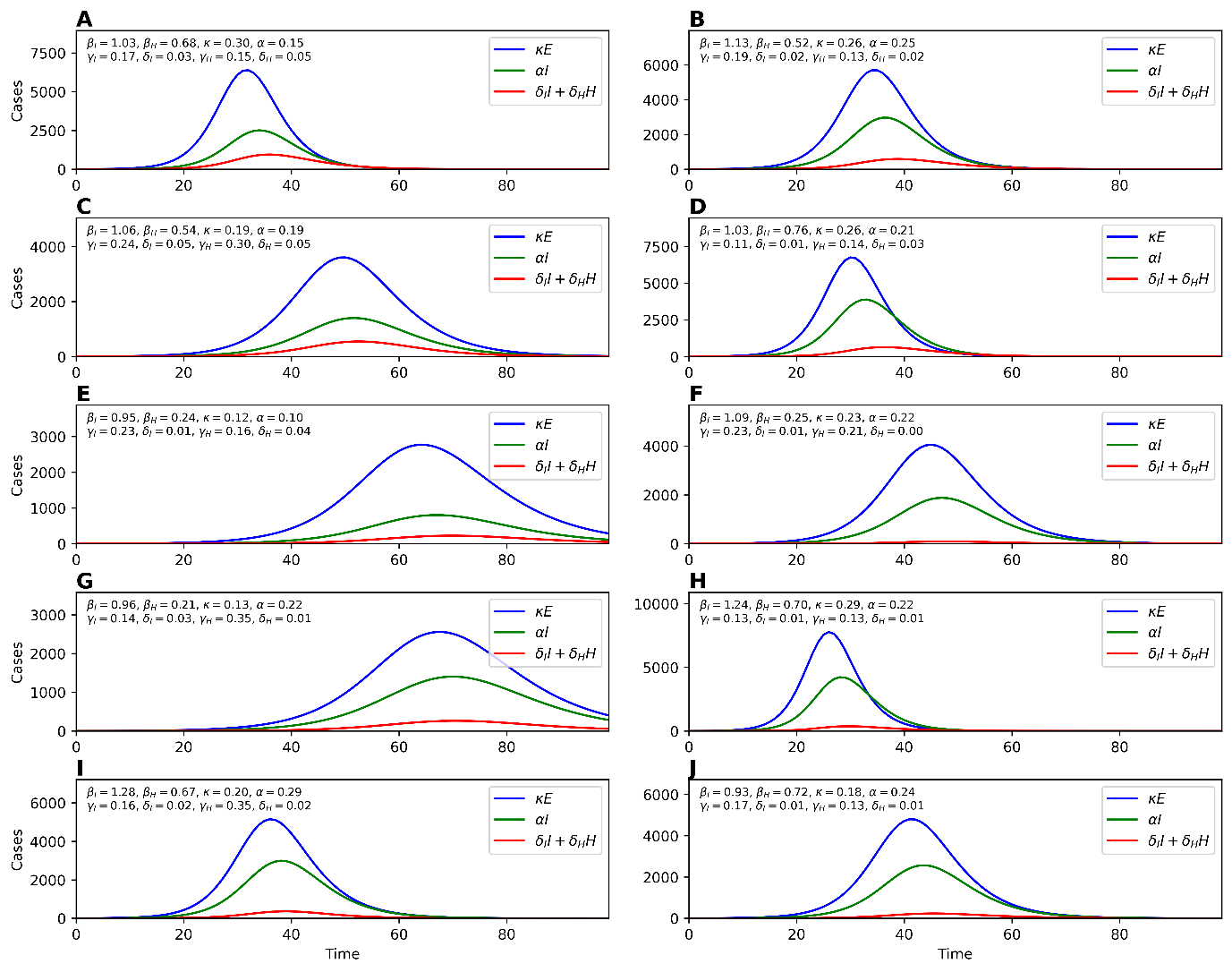
 **Fig 4 in S1 Text. Synthetic epidemic trajectories generated from the Ebola model using 10 distinct parameter sets.** **A–J** display one simulated trajectory with its corresponding parameter values ($\beta_{I}$, $\beta_{H}$, $\kappa$, $\alpha$, $\gamma_{I}$, $\delta_{I}$, $\gamma_{H}$, and $\delta_{H}$) indicated in the legend.


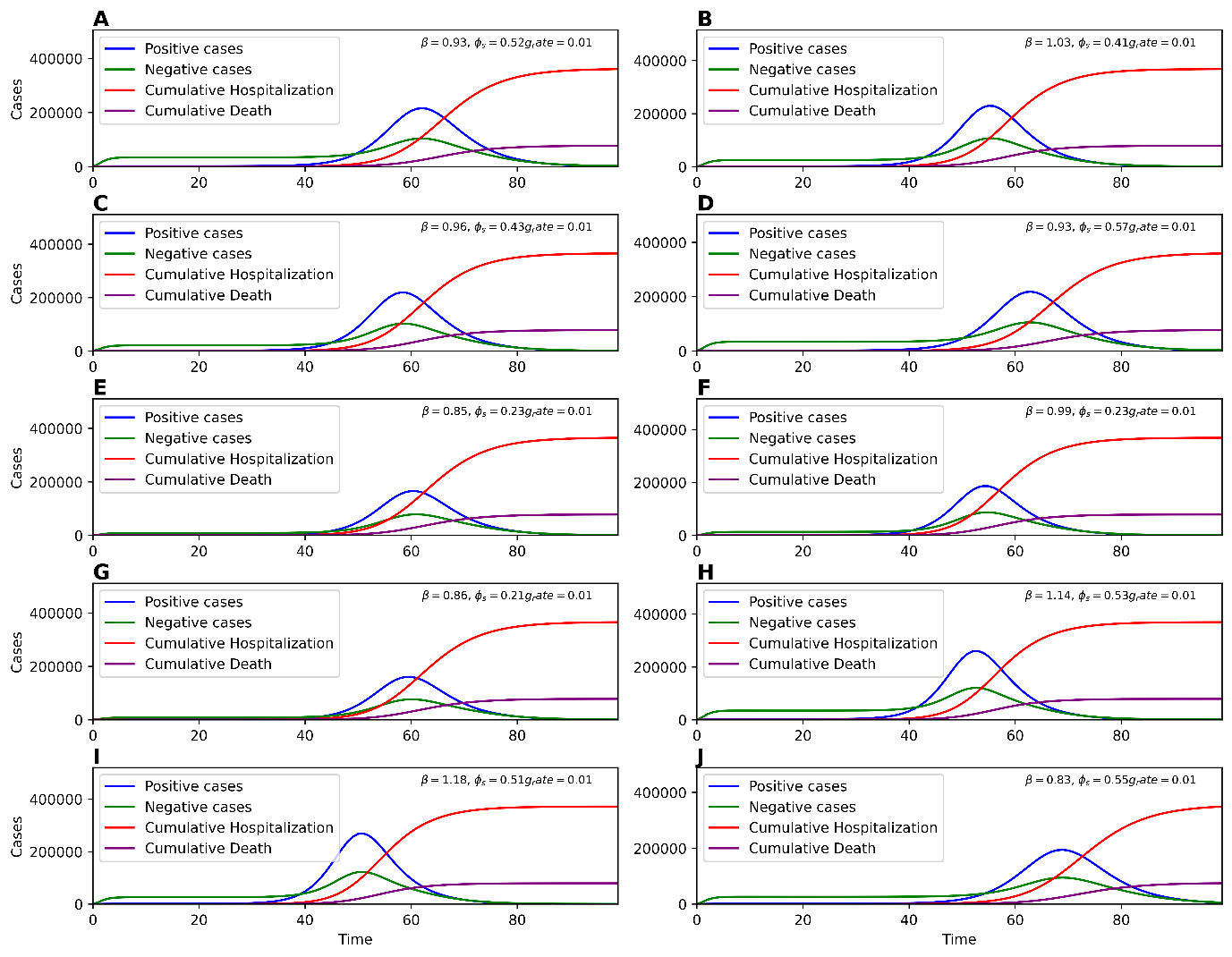


**Fig 5 in S1 Text. Synthetic epidemic trajectories generated from the SIRTEM model using 10 distinct parameter sets.** **A–J** display one simulated trajectory with its corresponding parameter values ($\beta$, $\phi_{s}$, and $g$) indicated in the legend.

**
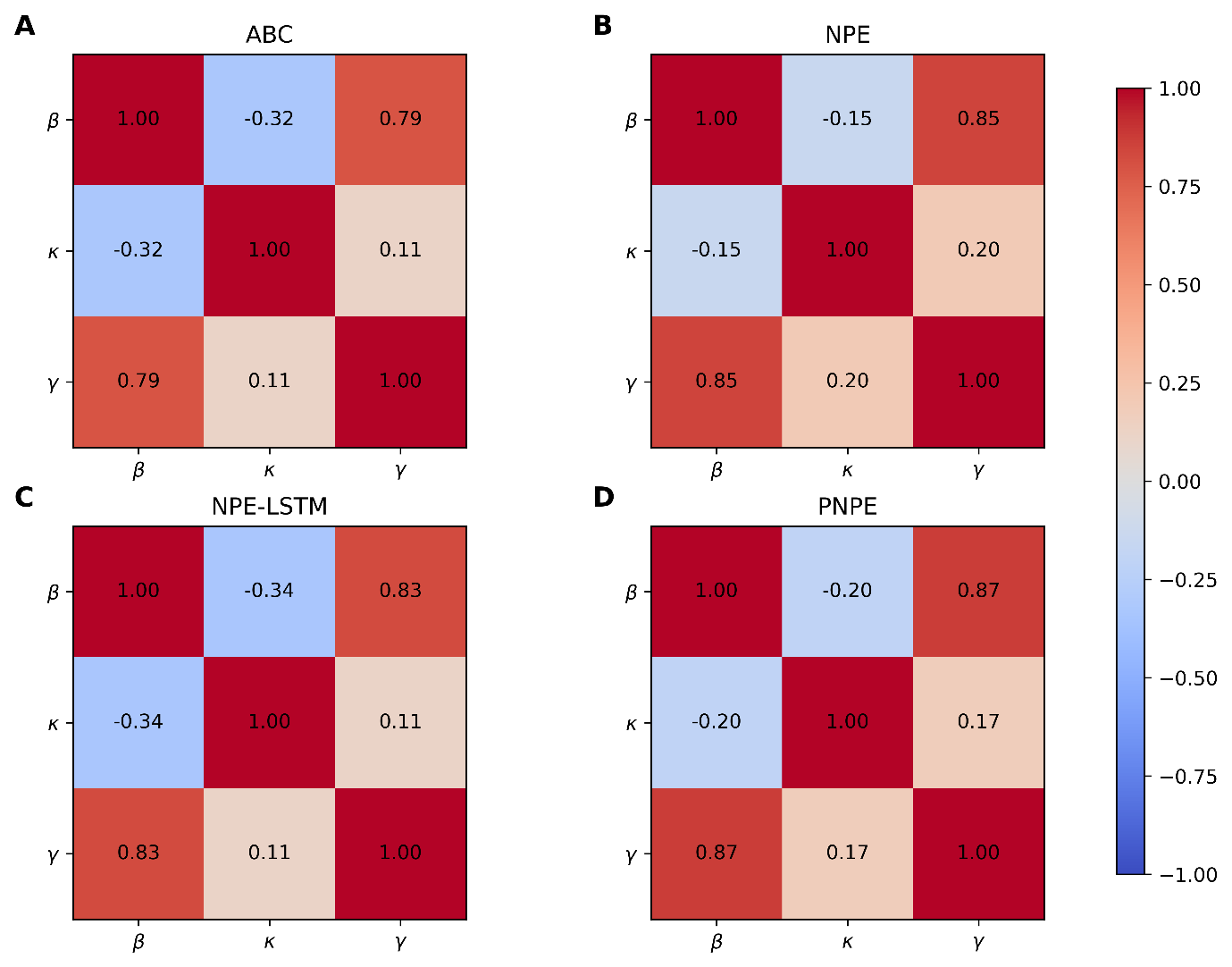
**

**Fig 6 in S1 Text. Posterior correlation heatmaps for ABC, NPE, NPE-LSTM, and PNPE.** The values indicate the Pearson correlation coefficients ($\rho$) between parameters ($\beta$, $\kappa$, and $\gamma$), averaged across 10 independent test sets.

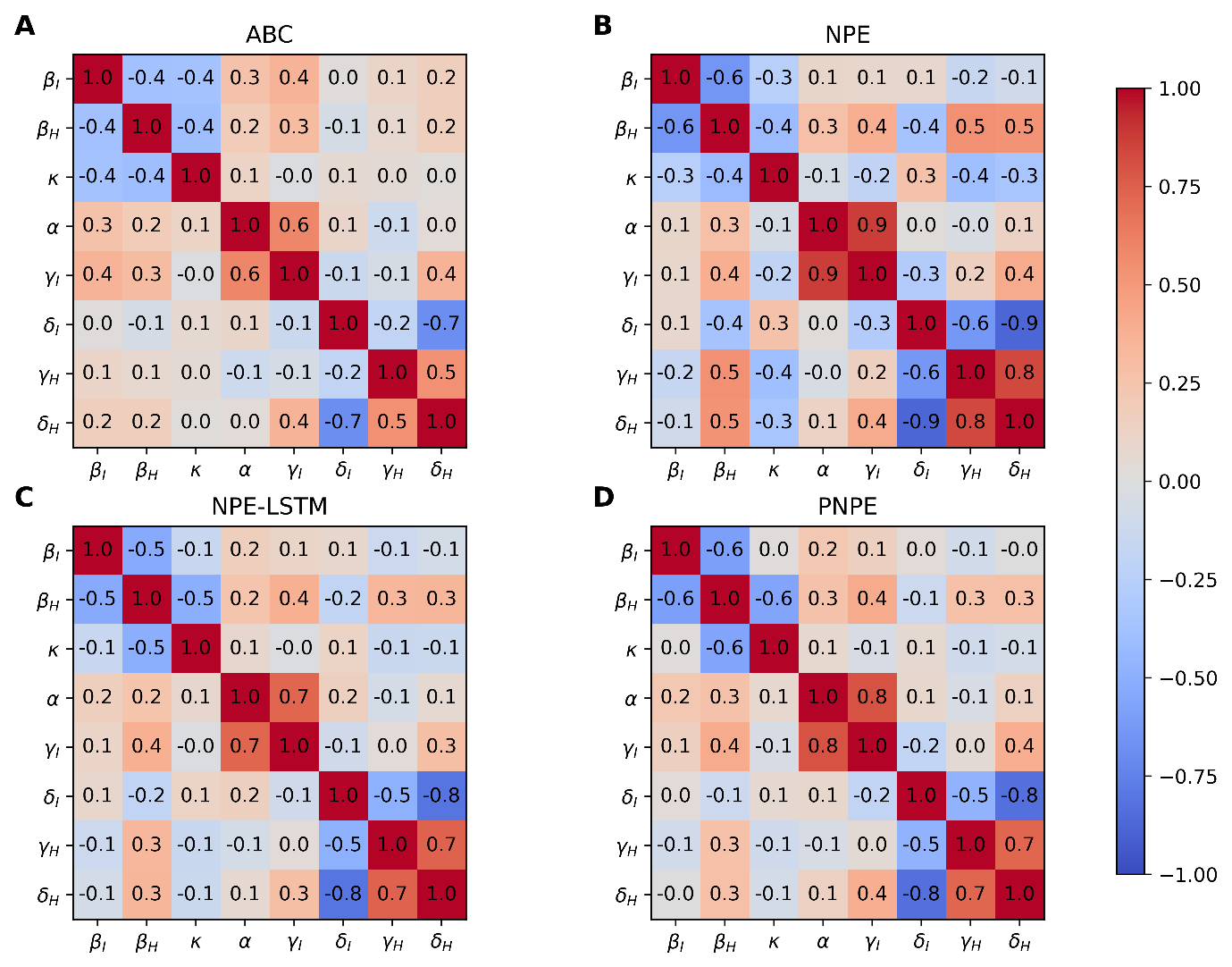


**Fig 7 in S1 Text. Posterior correlation heatmaps for ABC, NPE, NPE-LSTM, and PNPE.** The values indicate the Pearson correlation coefficients ($\rho$) between parameters ($\beta_{I}, \beta_{H}, \kappa, \alpha, \gamma_{I}, \delta_{I},\gamma_{H}$ and $\delta_{H}$) averaged across 10 independent test sets.
